# Myotubularin-related protein 7 activates peroxisome proliferator-activated receptor-gamma

**DOI:** 10.1038/s41389-020-0238-8

**Published:** 2020-06-10

**Authors:** Philip Weidner, Michaela Söhn, Torsten Schroeder, Laura Helm, Veronika Hauber, Tobias Gutting, Johannes Betge, Christoph Röcken, Florian N. Rohrbacher, Vijaya R. Pattabiraman, Jeffrey W. Bode, Rony Seger, Daniel Saar, Ariane Nunes-Alves, Rebecca C. Wade, Matthias P. A. Ebert, Elke Burgermeister

**Affiliations:** 1 Department of Medicine II, Medical Faculty Mannheim, Heidelberg University, Mannheim, Germany; 2 Department of Pathology, Christian-Albrechts University, Kiel, Germany; 3 Department of Organic Chemistry, ETH Zürich, Zürich, Switzerland; 4 Department of Biological Regulation, Weizmann Institute of Science, Rehovot, Israel; 5 Heidelberg Institute for Theoretical Studies (HITS), Heidelberg, Germany; 6 Zentrum für Molekulare Biologie der Universität Heidelberg (ZMBH), Heidelberg, Germany; 7 Interdisciplinary Center for Scientific Computing (IWR), Heidelberg University, Heidelberg, Germany

**Keywords:** Phosphorylation, Target identification, Phosphoinositol signalling, Colorectal cancer

## Abstract

Peroxisome proliferator-activated receptor-gamma (PPARγ) is a transcription factor drugable by agonists approved for treatment of type 2 diabetes, but also inhibits carcinogenesis and cell proliferation in vivo. Activating mutations in the Kirsten rat sarcoma viral oncogene homologue (*KRAS)* gene mitigate these beneficial effects by promoting a negative feedback-loop comprising extracellular signal-regulated kinase 1/2 (ERK1/2) and mitogen-activated kinase kinase 1/2 (MEK1/2)-dependent inactivation of PPARγ. To overcome this inhibitory mechanism, we searched for novel post-translational regulators of PPARγ. Phosphoinositide phosphatase *Myotubularin-Related-Protein-7* (MTMR7) was identified as cytosolic interaction partner of PPARγ. Synthetic peptides were designed resembling the regulatory coiled-coil (CC) domain of MTMR7, and their activities studied in human cancer cell lines and C57BL6/J mice. MTMR7 formed a complex with PPARγ and increased its transcriptional activity by inhibiting ERK1/2-dependent phosphorylation of PPARγ. MTMR7-CC peptides mimicked PPARγ-activation in vitro and in vivo due to LXXLL motifs in the CC domain. Molecular dynamics simulations and docking predicted that peptides interact with the steroid receptor coactivator 1 (SRC1)-binding site of PPARγ. Thus, MTMR7 is a positive regulator of PPARγ, and its mimicry by synthetic peptides overcomes inhibitory mechanisms active in cancer cells possibly contributing to the failure of clinical studies targeting PPARγ.

## Introduction

The nuclear transcription factor PPARγ has been established as a target in type 2 diabetes for many years. In addition to its lipid lowering and insulin sensitizing properties, pharmacological activation of PPARγ shows benefits in malignant and inflammatory human diseases^1–3^, and inhibition of RAS-ERK1/2 signalling was observed in (pre)clinical models^4–6^. It is the latter characteristic that renders the nuclear receptor a promising target in gastrointestinal tumours with frequent, activating mutations in the RAS-ERK1/2 signalling cascade, e.g. in colorectal cancer (CRC)^7^. In this setting, mutations in *RAS* genes are a major obstacle for effective treatment in advanced disease^8^, and new drugable targets which inhibit RAS-ERK1/2 signalling are needed^9^. However, serious adverse effects limit the long-term monotherapy with PPARγ-ligands in metabolic diseases^10^. Nonetheless, combination with chemo- or biological therapies may offer novel strategies against cancer^6,11,12^ Clinical trials investigating the use of PPARγ-agonists have yet failed to show sufficient efficacy^13,14^.

One reason for this discrepancy between preclinical and clinical studies may rely on the complex regulation of PPARγ by the RAS-ERK1/2 signalling cascade, which has not been taken into account in any of the before mentioned trials: we^15,16^ and others^17,18^ demonstrated that downstream effectors of RAS inhibit PPARγ, e.g. by ERK1/2-dependent phosphorylation as well as by nuclear export and cytosolic sequestration through MEK1. In addition to this regulatory mechanism, off-target side effects of the first generations of PPARγ-agonists even resulted in an increased proliferation rate of tumour and vascular cells, as they involve PPARγ-receptor independent (“non-genomic”) activation of RAS^19^ and phosphoinositide 3-kinase (PI3K)^20^ signalling, especially at higher dosages.

We therefore hypothesized that the resulting decrease in nuclear transcriptional activity of PPARγ, due to its cytosolic sequestration in the presence of an active RAS cascade promotes its targeting to so far unknown cytosolic effectors. Therefore, unravelling novel effectors or modulators of PPARγ could be a promising approach to overcome this obstacle, especially concerning tumours harbouring activating mutations of *RAS* genes, which are primarily unresponsive to PPARγ activation.

In this context, we identified 76 kDa myotubularin-related protein 7 (MTMR7), a member of the myotubularin (MTM) family of lipid phosphatases, as a novel interaction partner of PPARγ. MTMs consist of N-terminal plextrin homology (PH), central protein tyrosine phosphatase (PTP), SET-interaction (SID) and C-terminal coiled-coil (CC) domains^21,22^. Homo- and heterodimerization between a catalytically active member of the family with an enzymatically inactive one, e.g. MTMR6/7/8 with MTMR9, is mediated via the CC domain resulting in an increased enzymatic activity^23^. For murine MTMR7, a truncated 54 kDa isoform has been described lacking this domain^24^. The active enzyme then dephosphorylates phosphatidyl-inositol-3-monophosphate (PI(3)P) and -3,5-bisphosphate (PI(3,5)P_2_). MTMs are membrane-bound and localize to endosomes, with the exception of MTMR7, being present in a soluble form in the cytoplasm^24^ using free inositol-1,3-bisphosphate (Ins(1,3)P_2_) as a substrate.

In addition to the previously reported expression of MTMR7 in brain, muscle, liver and kidney^24^, we detected MTMR7 in the gastrointestinal tract^25^. In contrast to other MTMs, characterized as “survival phosphatases”^21,22^, we demonstrated that MTMR7 reduces proliferation of CRC cells in vitro, even in the presence of activating mutations of *KRAS* and active insulin signalling, due to inhibition of both RAS-ERK1/2 and PI3K-AKT-mTOR signalling^25^.

In the present study, we describe a novel regulatory mechanism of PPARγ which augments its transcriptional activity via its interaction with MTMR7. In addition, we offer new insights into the subcellular distribution of MTMR7 in response to external stimuli and identified the CC domain of MTMR7, by designing and modifying a peptide resembling this domain, as a potential novel pharmacological activator of PPARγ in vitro and in vivo.

## Results

### MTMR7 is a cytosolic binding partner of PPARγ

**Fig. 1:**
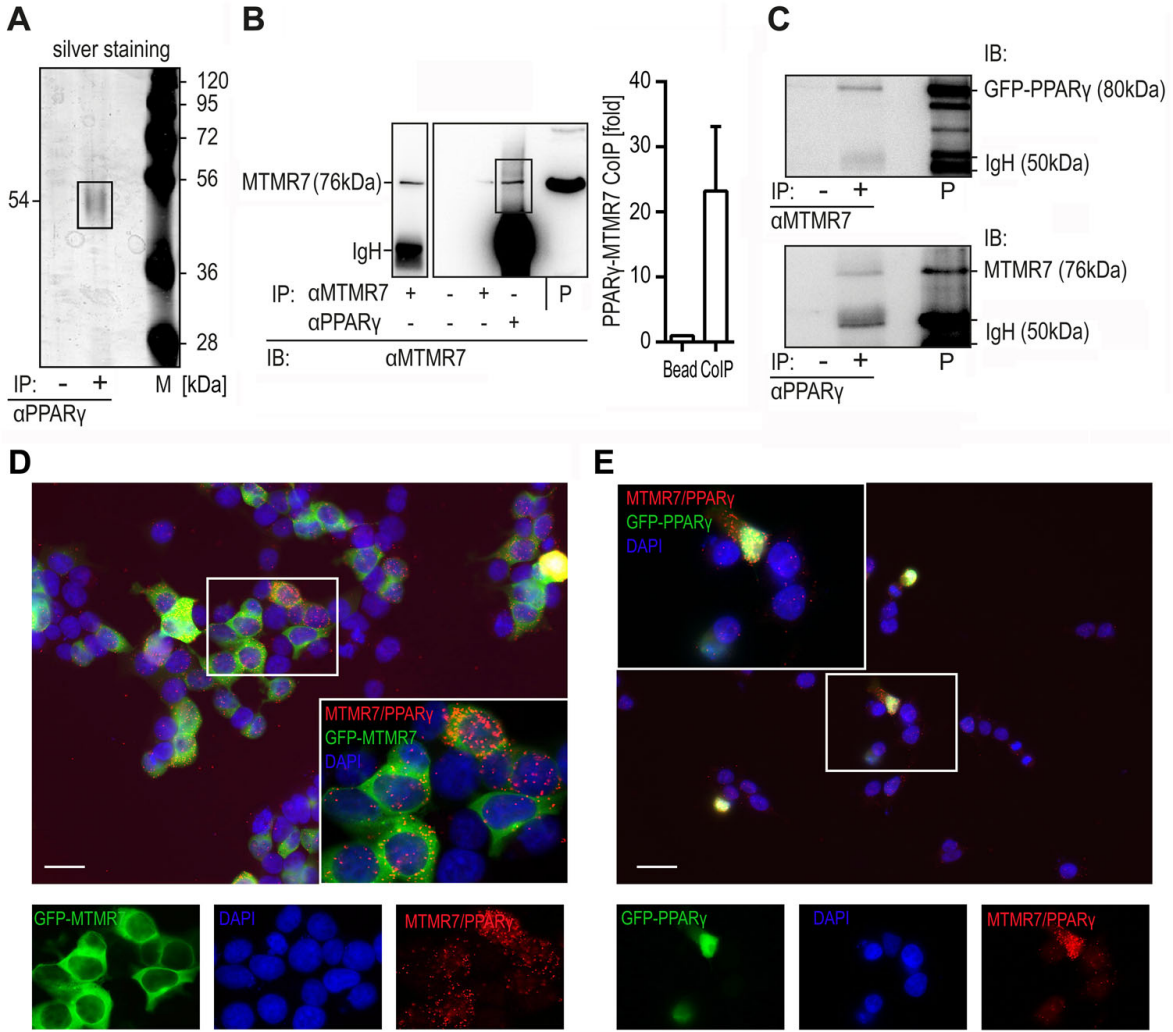
Identification of MTMR7 as a cytosolic interaction partner of PPARγ. **a** Coimmunoprecipitation (CoIP) was performed on cytosolic lysates of SW480 cells using PPARγ Ab (#7196) or IgG (bead control) followed by detection of precipitated bands by silver staining. MALDI-MS sequencing of a 54 kDa band precipitated by PPARγ Ab contained peptides from human MTMR7 (Tab. S1). M = marker. **b** CoIP of endogenous proteins in cytosolic lysates of HCT116 cells using PPARγ (lane 4: #7196) or MTMR7 (lane 1: #121222; lane 3: #51145) Abs for IP and IB, respectively. Representative gels and quantitative analyses are shown. Absolute O.D. values from bands in gels are shown as -fold ± S.E. compared with bead control (*n* = 3 replicates). P = input. **c** CoIP of ectopic proteins. HEK293T cells were transiently co-transfected with GFP-PPARγ and MTMR7 (FL) expression plasmids for 24 h. CoIP was performed on cytosolic lysates using PPARγ (#7196) or MTMR7 (#121222) Abs, respectively. Representative gels are shown. P = input. **d**, **e** Proximity ligation assay (PLA). HEK293T cells were co-transfected with untagged PPARγ and GFP-MTMR7 FL (**d**) or untagged MTMR7 FL and GFP-PPARγ (**e**) expression plasmids for 24 h, respectively. Immunofluorescence microscopy was performed using PPARγ (#2435) (**d**), MTMR7 (#121222) (**e**) and GFP Abs. Representative images are shown. Scale bar = 20 µm. Colour legend: red = PPARγ/MTMR7 complex; green = GFP; blue = nuclei (DAPI); original magnification ×630.

In cancer cells with constitutive activation of RAS-ERK1/2 signalling, PPARγ can be translocated from the nucleus to the cytosol by a previously described MEK1-dependent export mechanism^15,26^. However, the function of cytosolic PPARγ is unknown. To identify novel binding partners which may act as regulators or effectors for cytosolic PPARγ, a matrix-assisted laser desorption/ionization mass spectrometry (MALDI-MS) protein interaction screen was performed in the aneuploid human CRC cell line SW480, which has mutated alleles of the *KRASG12V* gene^27^ and a high amount of extra-nuclear PPARγ. SW480 cells were disrupted by hypotonic lysis without detergents, a procedure which extracts cytosolic proteins. Coimmunoprecipitation (CoIP) was then performed with PPARγ antibody (Ab) or control IgG followed by detection of precipitated bands by silver staining. Peptides within a band of 54 kDa were coprecipitated by the PPARγ Ab and identified to correspond to human MTMR7 by MALDI-MS sequencing (Fig. 1a; Tab. S1). Most of the peptides covered the internal and C-terminal part of the enzyme, including the catalytic phosphatase, SID and CC domains.

For detection of the cytosolic complex between endogenous MTMR7 and PPARγ, we resorted to HCT116, a human *KRASG13D* mutated CRC cell line which expresses high amounts of full-length (FL) MTMR7 (76 kDa) protein^25^. Subcellular fractionation (SCF) of normal cycling HCT116 cells was performed, and cytosolic lysates were immunoprecipitated with MTMR7 or PPARγ Ab-conjugated or unconjugated beads. Immunoblotting (IB) was performed with MTMR7 Ab (Fig. 1b). MTMR7 Abs were directed against the C-terminal region of the enzyme and pulled down a 76 kDa band corresponding to the FL protein.

For detection of ectopic proteins, we employed HEK293T cells which have low amounts of endogenous FL MTMR7 (76 kDa) protein^25^. Cells were transiently co-transfected with expression plasmids encoding for GFP-PPARγ and MTMR7 (FL) protein for 24 h. After SCF, CoIP from cytosolic lysates was performed using MTMR7 or PPARγ-Ab-conjugated beads. IB against MTMR7 detected an 80 kDa band, representing GFP-PPARγ after MTMR7 pulldown (Fig. 1c upper panel). For CoIP performed with PPARγ Ab-conjugated beads, a 76 kDa overexpressed band was detected by the MTMR7 Ab (Fig. 1c lower panel). To corroborate the findings on this interaction, proximity ligation assay (PLA) was conducted in HEK293T cells after a 24 h transfection with GFP-MTMR7 (FL) (Fig. 1d) or GFP-PPARγ (Fig. 1e) expression plasmids, respectively. Thereafter, cells were subjected to immunofluorescence imaging, and colocalization of MTMR7 and PPARγ proteins was visualized as pink dots. These data indicated that MTMR7 forms a complex with PPARγ.

### Nuclear translocation of MTMR7 in response to growth factors and PPARγ-agonist

**Fig. 2:**
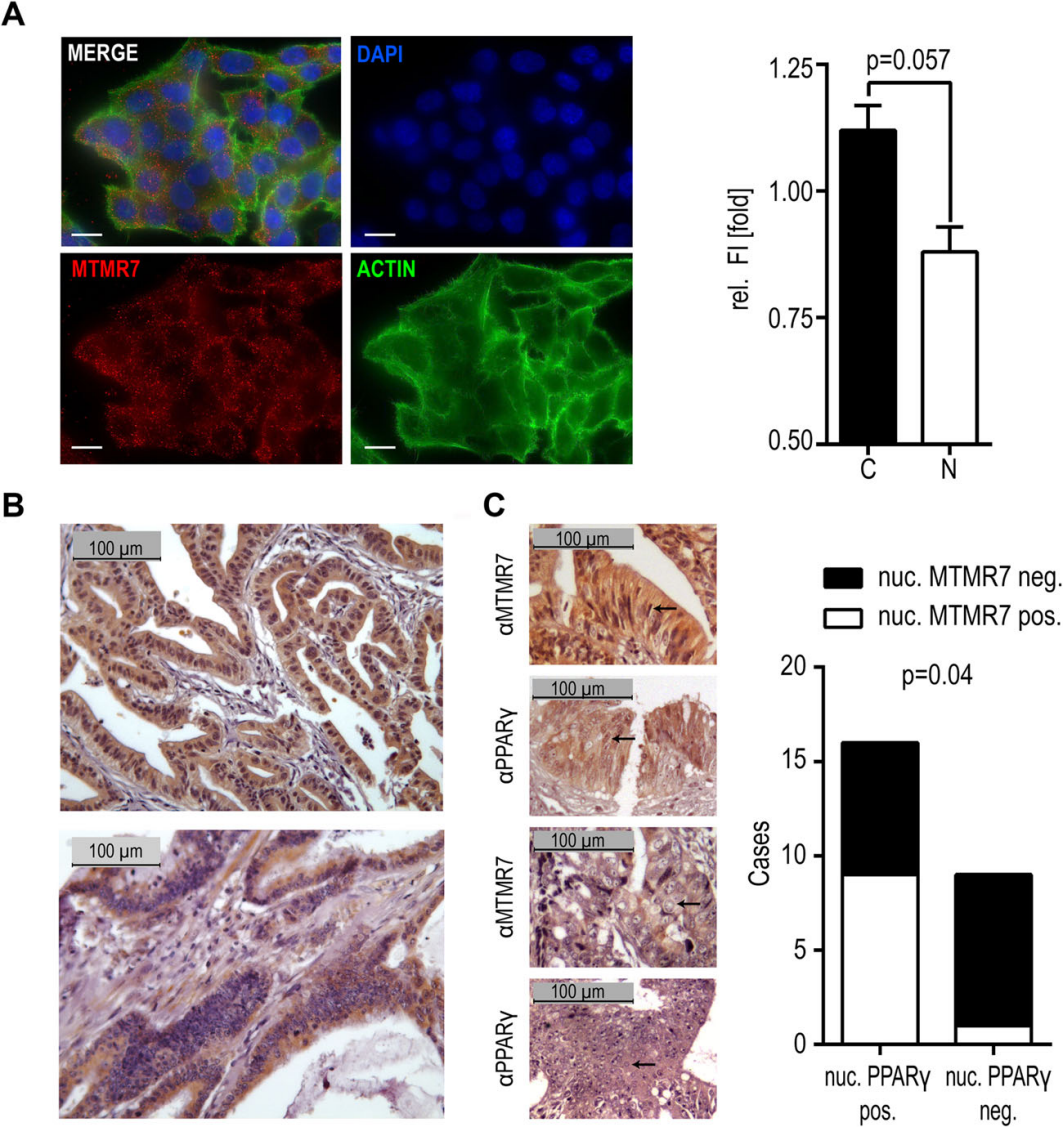
Subcellular distribution of MTMR7 protein variants. **a** Localisation of MTMR7. Immunofluorescence microscopy was performed using MTMR7 Ab (#121222) in HCT116 cells, expressing endogenous MTMR7 protein. Left: Representative images. Right: Quantification of nuclear (“N”) and cytoplasmatic (“C”) fluorescence intensity (FI) revealed a predominant cytoplasmatic localization of MTMR7. Data are -fold FI ± S.E. compared with control (*p* = 0.057 N vs. C, Mann–Whitney test, *n* = 4 replicates). Scale bar = 10 µm. Colour legend: red = MTMR7, green = actin (phalloidin), blue = nuclei (DAPI); original magnification ×630. **b** Localisation of MTMR7 in human CRC patients’ tissues (*n* = 67 cases). Immunohistochemistry (IHC) using MTMR7 Ab (#121222) revealed a subset of patients with positive nuclear MTMR7 staining (upper panel), whereas it was detectable mainly in the cytosol of other cases (lower panel). Representative images are shown; original magnification ×200. **c** Reduced nuclear localization of PPARγ is associated with increased cytosolic localization of MTMR7. IHC staining with MTMR7 (#121222) and PPARγ (#7196) Abs was performed on matched human CRC patients’ tissue specimens. Left: Representative images of cases with (upper two panels) or without (lower two panels) nuclear MTMR7 and PPARγ staining are shown; representative nuclei are marked by arrows. Right: Quantitative analyses evinced that lower numbers of PPARγ^+^ nuclei correlated with reduced MTMR7^+^ nuclei (*p* = 0.04, Fisher’s exact test, *n* = 67 cases). Reduction of nuclear PPARγ staining was defined as <30% positive nuclei.

To interrogate the subcellular distribution of MTMR7 and PPARγ, immunofluorescence microscopy was performed on normal cycling HCT116 cells expressing high levels of endogenous MTMR7 protein (Fig. 2a)^25^. In this setting, predominant cytosolic localization could be detected (*p* = 0.057 nuclear “N” vs. cytosolic “C”, Mann–Whitney test, *n* = 4 replicates). In addition, we evaluated formalin-fixed paraffin-embedded (FFPE) tissue specimens from CRC patients (*n* = 67 cases) with regard to MTMR7 expression and subcellular localization by means of immunohistochemistry (IHC). Of these patients, 41 (61%) showed a positive MTMR7 staining. In 19 (46%) of these MTMR7 positive cases, a nuclear staining was detected (Fig. 2b).

For 25 samples, an additional staining for PPARγ was observed, 16 (64%) of which exhibited a positive nuclear staining for PPARγ (Fig. 2c). Nuclear PPARγ staining was associated with nuclear localization of MTMR7 (*p* = 0.04, Fisher’s exact test, *n* = 67 patients).

**Fig. 3:**
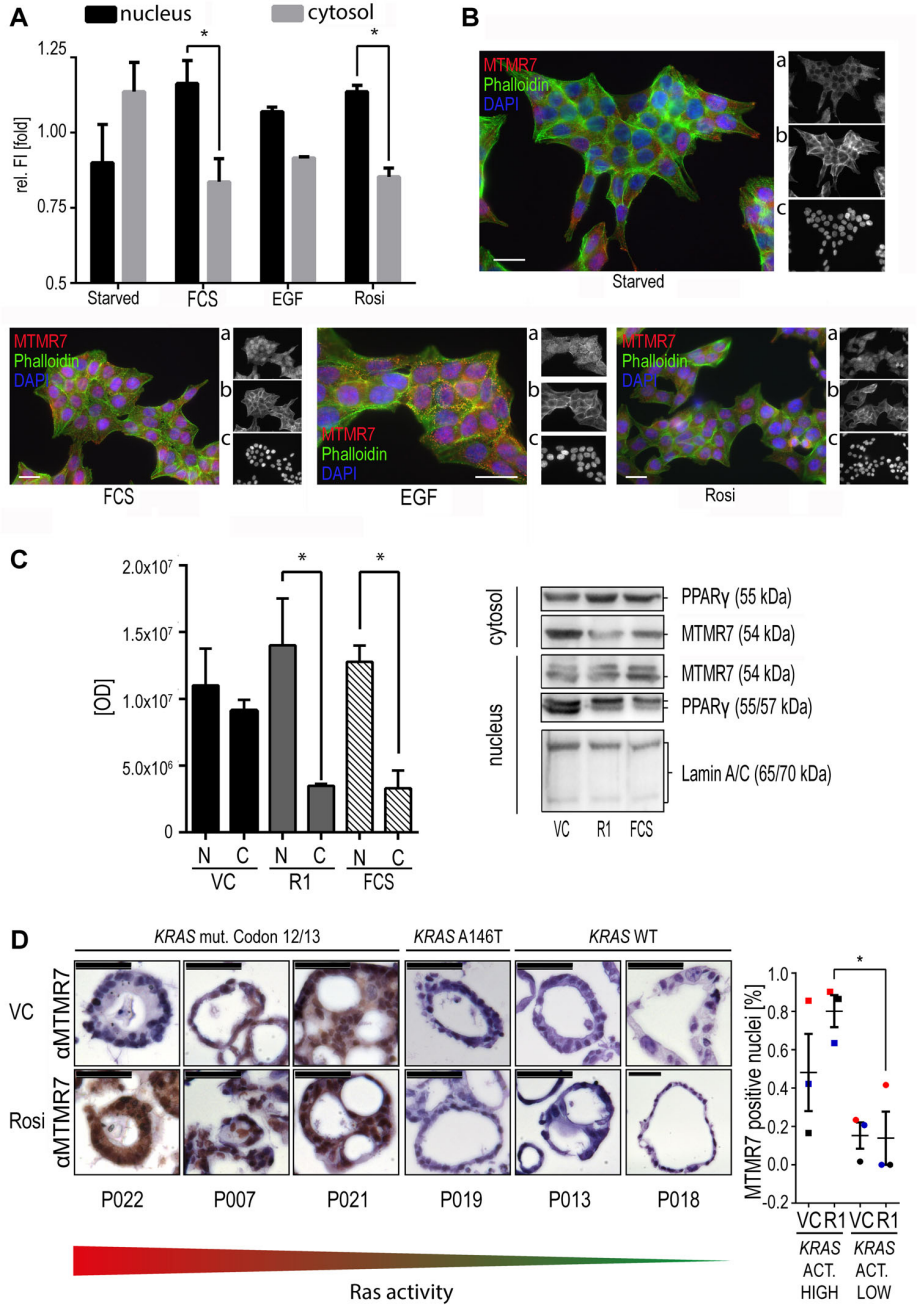
Nuclear translocation of MTMR7 in response to growth factors and PPARγ-agonist. **a** Immunfluorescence microscopy. HCT116 cells were serum-deprived for 16 h (“starved”) and subsequently stimulated with serum (20% v/v FCS), EGF (50 ng/ml) or rosi (1 μM) for 1 h, followed by fixation and staining of endogenous MTMR7 (Ab: #121222). FI signals from the nuclear and cytosolic compartments were normalized to the overall FI. Data are -fold FI ± S.E. (*p < 0.05 N vs. C, two-way ANOVA, Sidak’s multiple comparisons test, *n* = 3 replicates). While there was no difference in the distribution of endogenous MTMR7 in starved cells, 1 h stimulation with serum, EGF or rosi increased nuclear MTMR7 FI. **b** Representative images from (**a**) using Abs against MTMR7 (red, a), actin (phalloidin) (green, b), nuclei (DAPI) (blue, c). Scale bars = 20 µm. Original magnification: ×630. **c** Endogenous MTMR7 translocates into the nucleus in response to growth factors and PPARγ-agonist. HEK293T cells were serum-deprived for 16 h followed by incubation with vehicle control (VC; DMSO), rosi (R; 1 μM) or 20% (v/v) FCS for 1 h. Thereafter, cells were subjected to SCF and IB for MTMR7 (Ab: #150458). Representative gels and quantitative analyses. Mean O.D. values ± S.E. from bands in gels are shown (*p < 0.05 N vs. C, two-way ANOVA, Sidak’s multiple comparisons test, *n* = 3 replicates). C = cytoplasm; N = nucleus. **d** MTMR7 translocates into the nucleus in patient-derived organoids (PDOs). Cell lines from six different CRC patients were cultivated for 48 h in medium containing VC or 1 µM rosi (R1). Left panel: IHC against MTMR7 (Ab: #9406043) was performed, and 4 of 6 PDO lines showed a positive MTMR7 staining at baseline which increased upon rosi stimulation. In 2 of 3 PDO lines with activating *KRAS* mutations, MTMR7 staining intensity increased after rosi stimulation. Each PDO is labelled by its patient ID. The *KRAS*-mutation type is given above the representative images. Right panel: The percentage of MTMR7^+^ nuclei after rosi stimulation was higher in PDO lines with activating codon 12 and 13 mutations of *KRAS* (*p* = 0.016 high vs. low KRAS activity, two-way ANOVA, Holm–Sidak’s multiple comparisons test, *n* = 6 cases). Pre- and post-stimulation pairs are coded in the same colour.

To further study stimulation-dependent nuclear translocation of MTMR7, immunofluorescence microscopy was performed in HCT116 cells expressing high endogenous MTMR7 FL protein. Cells were serum-deprived for 16 h, followed by incubation with PPARγ-agonist rosiglitazone (rosi) (1 μM, R1), epidermal growth factor (EGF) (50 ng/ml) or foetal calf serum (FCS) (20% v/v) for 1 h (Fig. 3a, b). Upon stimulation with either FCS or rosi, an increase in the nuclear fluorescence intensity (FI) was detected (FCS *p* = 0.0173, R1 *p* = 0.0431), while there was a trend for a predominant cytoplasmic localization in starved cells (*p* = 0.11) (N vs. C, two-way ANOVA, Sidak’s multiple comparisons test, *n* = 3 replicates). Thus, endogenous MTMR7 translocates from the cytosol to the nucleus in response to serum or PPARγ-activation.

However, this method did not discriminate between FL and small isoforms of MTMR7^24,25^. We therefore studied subcellular localization in HEK293T cells which had low endogenous MTMR7 FL protein. Cells were starved for 16 h and then stimulated with serum or PPARγ-agonist, followed by SCF and IB against MTMR7 (Fig. 3c). Upon incubation with 20% (v/v) FCS or rosi (1 μM, R1), the endogenous, truncated MTMR7 isoform (54 kDa) accumulated in the nuclear fraction (N vs. C: FCS *p* = 0.0440, R1 *p* = 0.0008; two-way ANOVA, Sidak’s multiple comparisons test, *n* = 3 replicates). All Abs used in the present study recognized the C-terminal region of the MTMR7 FL protein (Tab. S2), indicative of a stimulus-dependent mobility of both isoforms.

To characterize the subcellular localization of MTMR7 in situ, we cultivated patient-derived organoids (PDOs) of four patients harbouring activating *KRAS* mutations and two patients with wild-type (WT) *KRAS*. The PDOs were incubated for 48 h in medium containing 1 µM rosi or vehicle control (VC, DMSO) (Fig. 3d): On baseline, 4 of the 6 PDOs showed a positive MTMR7 staining, 3 of which had an activating *KRAS* codon 12 or 13 mutation. Remarkably, the rosi-stimulated PDOs showed an increase in nuclear MTMR7. This effect was more pronounced in the PDO lines with the activating *KRAS* mutations of codon 12 or 13 as compared with *KRAS* WT PDO lines or the line with the A146T mutation (*p* = 0.016 high vs. low KRAS activity, two-way ANOVA, Holm–Sidak’s multiple comparisons test, *n* = 6 cases). Thus, PPARγ-agonist facilitates nuclear accumulation of MTMR7 in vitro and ex vivo in patient-derived cancer stem cells.

### MTMR7 increases nuclear transcriptional activity of PPARγ

**Fig. 4:**
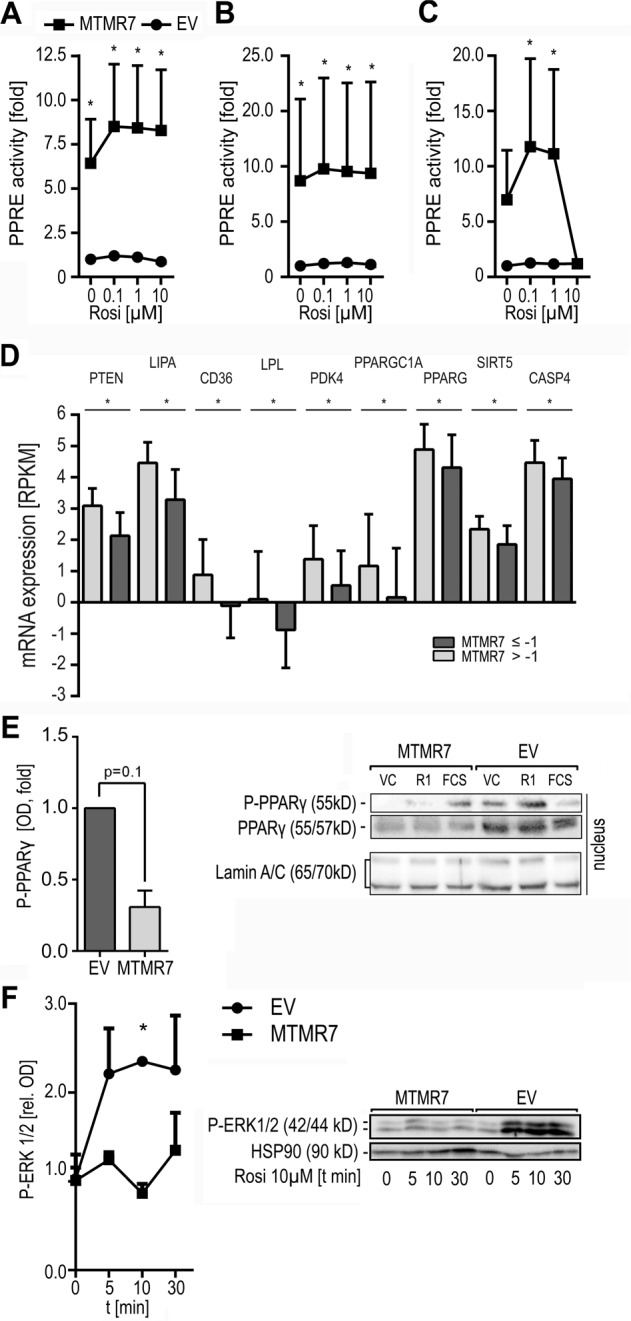
MTMR7 increases the transcriptional activity of PPARγ. **a**–**c** MTMR7 promotes PPARγ-dependent reporter gene expression. HCT116 (**a**), SW480 (**b**) and HEK293T (**c**) cells were transfected with empty vector (EV) or MTMR7 FL expression plasmid, respectively, together with PPARγ-reporter plasmid (PPRE-luc) for 24 h before stimulation with rosi (0–1 µM) for additional 24 h. Luciferase activity was measured in total cell lysates, normalised to protein content and calculated as -fold ± S.E. compared with control (*p < 0.05 EV vs. MTMR7, two-way ANOVA Sidak’s multiple comparisons test; replicates: HCT116 *n* = 12; SW480 *n* = 6; HEK293T *n* = 12). **d** Downregulation of *MTMR7* mRNA is associated with decreased expression of PPARγ target genes. In silico analysis using the Colorectal Adenocarcinoma (TCGA, Nature 2012) dataset provided by the cBioPortal database for Cancer Genomics revealed a downregulation ≥1 S.D. in 38 of 182 CRC patients analysed. This downregulation was associated with reduced “Reads per kilo base per million mapped reads” (RPKM) counts of PPARγ target genes. Data are means ± S.D. and significances (*p < 0.05 high vs. low *MTMR7* mRNA) as disclosed by the provider. **e** MTMR7 reduces inhibitory phosphorylation on serine 82/84 on PPARγ1. HEK293T cells were transfected with MTMR7 (FL) for 6 h, followed by serum-deprival for 16 h and subsequent stimulation with vehicle (VC, DMSO), rosi (1 μM, R1) or 20 % (v/v) FCS for 60 min, followed by SCF. IB of unphosphorylated and phosphorylated PPARγ was performed. Data are absolute O.D. values calculated as -fold ± S.E. compared with EV control (*p* = 0.1 EV vs. MTRM7, Mann–Whitney test, *n* = 3 replicates). **f**, MTMR7 abrogates rosi-mediated activation of ERK1/2. HCT116 cells were transfected with MTMR7 FL plasmid for 6 h, followed by serum-deprival for 16 h and stimulation with rosi (10 µM) for the times indicated (min). IB on total cell lysates detected reduced ERK1/2 phosphorylation in presence of MTMR7. Data are absolute O.D. values normalized to HSP90 calculated as -fold ± S.E. (*p* = 0.0449, Kruskal–Wallis test, subgroup analysis: 10 min, *p* = 0.0392 EV vs. MTMR7, Dunn’s multiple comparisons test; *n* = 3 replicates).

To assess, whether MTMR7 alters the transcriptional activity of PPARγ, we performed reporter gene assays using a luciferase plasmid with a PPARγ-responsive enhancer element (PPRE) in front of a basal promoter. HCT116, SW480 and HEK293T cells were transfected with empty vector (EV) or MTMR7 (FL) expression plasmid followed by stimulation with rosi (0.1–10 μM) for 48 h (Fig. 4a–c). MTMR7 overexpression increased basal (HCT116 & SW480: *p* < 0.0001) and ligand-dependent (HCT116 & SW480: *p* < 0.0001; HEK293T: 1 μM: *p* = 0.0295, 10 µM: *p* = 0.0425) reporter gene activity compared with EV control (replicates: HCT116: *n* = 12; SW480: *n* = 6; HEK293T: *n* = 12; two-way ANOVA, Sidak’s multiple comparisons test, EV vs. MTMR7).

To explore whether these results correlate with increased PPARγ-target gene expression, in silico analysis using the cBioportal database of cancer genomics was conducted^28^. *MTMR7* mRNA expression ≤1 S.D. of the mean positively correlated with a reduced expression of a broad set of PPARγ-target genes in the CRC dataset provided by *TCGA Nature 2012* (Fig. 4d). This finding was reproduced in the dataset provided by the *Pancancer Atlas* (not shown).

To elucidate a possible mechanism underlying the observed increased PPARγ-activity in presence of MTMR7, we performed SCF followed by IB for phosphorylated PPARγ, using an Ab specific for the bona fide ERK1/2-phosphorylation site at serine 82/84. To this end, HEK293T cells were transfected with EV or MTMR7 (FL) and treated with vehicle control (VC), 20% (v/v) FCS or 1 μM rosi (R1) for 24 h, respectively (Fig. 4e). After MTMR7 overexpression, a trend for reduced amount of nuclear, phosphorylated (i.e. inactivated) PPARγ was detectable. Hence, MTMR7 seems to increase PPARγ-activity indirectly by reducing its inhibitory phosphorylation through the RAS-MEK1/2-ERK1/2 pathway^16^.

### MTMR7 inhibits PPARγ-agonist-mediated ERK1/2 activation

Since at least some of the off-target side effects of rosi (and related glitazones) may be mediated by the aberrant activation of MEK1/2-ERK1/2 signalling^29,30^, we investigated the effect of MTMR7 overexpression on rosi-mediated ERK1/2 phosphorylation. To this end, HCT116 cells were transiently transfected with EV or MTMR7 (FL), serum-deprived for 16 h and subsequently incubated with 10 µM rosi, a concentration higher than its IC_50_ and shown to stimulate ERK1/2^30^. This effect was abrogated by overexpression of MTMR7, leading to reduced amounts of phosphorylated ERK1/2 (*p* = 0.0449, Kruskal–Wallis test), particularly after 10 min of rosi stimulation (*p* = 0.0392 EV vs. MTMR7, Dunn’s multiple comparisons test) (*n* = 3 replicates) (Fig. 4f). A similar result was shown for SW480 cells (Supplementary Fig. 1A).

### Design of MTMR7-CC mimicry peptides

**Fig. 5:**
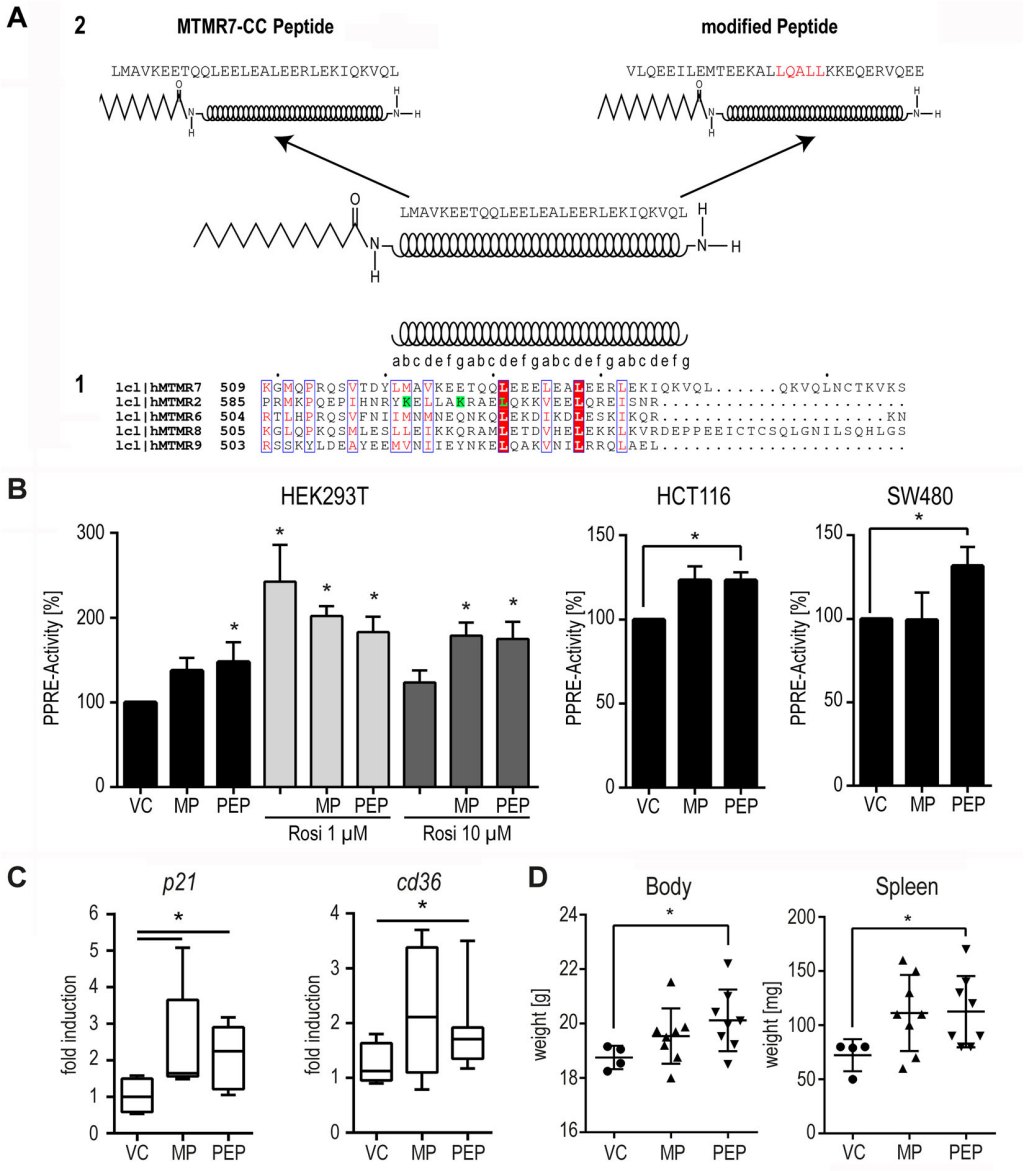
Design of MTMR7-CC mimicry peptides which activate PPARγ in vitro and in vivo. **a**, **1**: Alignment of the amino acid (aa) sequences of the coiled-coil (CC) domains of MTMR7, 2, 6, 8 and 9. The heptad repeat is denoted with the letters a–g, with the leucines of MTMR2 residing in the d position. Residues of MTMR2 required for heterodimerization are marked green^32^. This figure was drawn using ESPript (http://espript.ibcp.fr). **a**, **2**: MTMR7 harbours a 30 aa sequence of MTMR7 with high coil-forming capacity. The N-terminus was modified by myristoylation, the C-terminus was amidated. A modified peptide consisting of the same aa sequence in a scrambled order, but with a PPARγ coactivator LXXLL motif, was designed as control. **b** MTMR7-CC peptides promote transcriptional activity of PPARγ. HEK293T, HCT116 and SW480 cells were transfected with PPARγ-luciferase reporter plasmid (PPRE-luc) for 24 h before incubation with 1 μM of a peptide mimicking the coiled-coil (CC) domain of MTMR7 (PEP) and a modified peptide (MP) with a scrambled α-helix composition but a preserved LXXLL coactivator motif in presence or absence of rosi (1 & 10 μM) for additional 24 h. Luciferase activity normalised to protein content was calculated as % ± S.E. compared with vehicle control (*p < 0.05 vs. VC, HEK293T: two-way ANOVA, Holm–Sidak’s multiple comparisons test, *n* = 7 replicates; HCT116: one-sample *t* test, *n* = 3 replicates, SW480: one-sample *t* test; *n* = 5 replicates). **c** PEP and MP activate PPARγ-target genes in vivo. C57BL6/J mice (pCEA-SV40-Tag) were treated for 14 days with MP, PEP (both at 30 mg/kg*d, four times a week) or vehicle control (VC, DMSO) by intraperitoneal injection, respectively. RT-qPCRs detecting PPARγ-target genes were performed on RNA extracted from snap-frozen whole tissue samples derived from the distal colon. MP and PEP treatment increased *P21*^*(Cip1/Waf1)*^ and *Cd36* mRNA expression. CT-values were normalized to *B2m* and calculated as -fold ± S.E. and compared with controls (*p < 0.05 vs. VC, Mann–Whitney test, VC: *n* = 4, MP: *n* = 8, PEP: *n* = 8 mice per group). **d** PEP and MP increase spleen weight and induce body weight gain, thereby mimicking a rosi-like phenotype^60^: pCEA-SV40-Tag mice were treated as above. Body and spleen weights were measured after 14 days of therapy. Data are means ± S.E. compared with controls (*p < 0.05 vs. VC, Mann–Whitney test, VC: *n* = 4, MP: *n* = 8, PEP: *n* = 8 mice per group).

The main regulatory mechanism of catalytically active MTMs is the formation of homo- or heterodimers with partner MTMs and effector proteins by means of the C-terminal coiled-coil (CC) domain^31^. We therefore hypothesized that the CC domain of MTMR7 is the region of the protein responsible for the interaction with PPARγ. To test this idea, we designed a synthetic peptide to substitute for the MTMR7 FL protein. There is no crystal structure of the MTMR7’s CC domain or any other MTM available. However, Kim et al. identified and characterized the CC domain of MTMR2^32^. Performing multiple alignment analysis of the amino acid (aa) sequences of MTMR7, MTMR2, MTMR6, MTMR8 and MTMR9, we identified a stretch of 30 amino acids at the C-terminus of MTMR7 (Fig. 5a1).

This leucine-rich region showed a high coil-forming capacity using NCOILS version 1.0 (ExPASy; Supplementary Fig. 1B). Therefore, we used this region as a basis for the design of a mimicry peptide (PEP) of the MTMR7-CC. Pepwheel, provided by EMBOSS, predicted the selected sequence to form an amphipathic α-helix (Supplementary Fig. 1C). In addition, a modified peptide (MP), consisting of the same amino acid residues in a scrambled order, but containing a canonical LXXLL coactivator motif for nuclear receptors^33^ (including PPARγ) was designed (Fig. 5a2). Both peptides were myristoylated at the N-terminus and amidated at the C-terminus to enhance uptake into cells and minimize proteolysis.

### MTMR7 mimicry peptides activate PPARγ in vivo and in vitro

To determine the effect of the peptides on the transcriptional activity of PPARγ, HEK293T cells were transfected with the PPRE-luciferase reporter plasmid for 24 h. Thereafter, treatment with vehicle control (VC), MP or PEP (both at 1 µM) was performed in presence or absence of rosi (1 and 10 μM). As for overexpression of MTMR7 FL protein, treatment with both peptides increased the transcriptional activity of PPARγ (Fig. 5b, *p < 0.05 vs. VC, two-way ANOVA, Holm–Sidak’s multiple comparisons test, *n* = 7 replicates). A similar effect was observed in HCT116 (*n* = 3) and SW480 (*n* = 5) replicates (*p < 0.05 vs. VC, one-sample *t* test). Treatment with concentrations of rosi higher than its IC_50_ (>1 µM) reduced PPARγ activity, presumably due to a “non-genomic” activation of the MEK1/2-ERK1/2-pathway and subsequent phosphorylation and inactivation of PPARγ^16^. Of note, exposure of cells to MP and PEP reversed this effect: In the presence of both peptides, increased PPARγ activity was detectable even after treatment with 10 μM rosi (Fig. 5b, *p < 0.05 vs. VC, two-way ANOVA, Holm–Sidak’s multiple comparisons test, replicates: HEK293T: *n* = 7, HCT116: *n* = 3, SW480: *n* = 5). To address the question whether this in vitro efficacy could be translated into the in vivo situation, C57BL6/J mice (pCEA-SV40-Tag)^34^ were treated for 14 days with MP, PEP (both at 30 mg/kg*d) or vehicle control (VC) by intraperitoneal injection (four times per week), respectively. RT-qPCRs (Tab. S2) detecting exemplary PPARγ-target genes were performed on total RNA extracted from snap-frozen whole tissue samples derived from the distal colon (Fig. 5c). *P21*^*(Cip1/Waf1)*^ and *Cd36* mRNAs were increased in animals treated with either of the peptides (VC: *n* = 4 mice, MP: *n* = 8 mice, PEP: *n* = 8 mice, *p < 0.05 vs. VC, Mann–Whitney test). This effect was accompanied by an increase in body weight (VC vs. PEP: *p* = 0.0112; VC vs. MP: *p* = 0.1, Mann–Whitney test) and spleen weight (VC vs. PEP: *p* = 0.0182; VC vs. MP: *p* = 0.0639, Mann–Whitney test) (Fig. 5d). Taken together, the MTMR7-CC mimicry peptides activated PPARγ in vitro and in vivo.

### Prediction of peptide/PPARγ structures in silico

To gain a better understanding of the potential molecular mechanism of the mimicry peptide mediated activation of PPARγ, a computational approach was employed to generate plausible models of the peptide/PPARγ complexes. Both peptides (PEP and MP) were predicted to form α-helical secondary structures by the web servers PredictProtein^35^ and Agadir^36^ (Supplementary Figs. 2, 3). Therefore, they were both modelled as α-helices, but the terminal myristoylation and the amide caps were omitted.

**Fig. 6:**
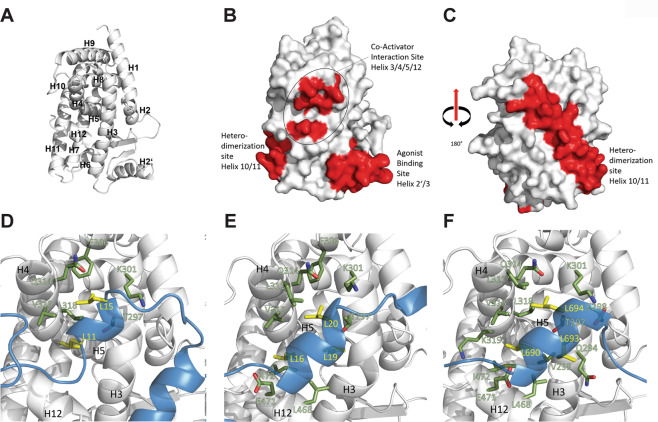
Prediction of peptide/PPARγ structures in silico. **a** Cartoon representation of PPARγ with numbered helices. PDB code: 1fm9^45^. **b**, **c** Surface representation of PPARγ. Possible interaction sites coloured in red. The agonist-binding site, formed by residues of helices 3 and 2ʹ, was predicted as a possible interaction site by the web server meta-PPISP (http://pipe.scs.fsu.edu/meta-ppisp.html, ref. ^37^). The coactivator interaction site, including residues from helices 3, 4, 5 and 12, was predicted by the web server PINTS (http://www.russelllab.org/cgi-bin/tools/pints.pl, ref. ^42^). Helix 10/11, the heterodimerization site, was predicted by coiled-coil predicting web servers DeepCoil (https://toolkit.tuebingen.mpg.de/#/tools/deepcoil, ref. ^41^) and Waggawagga (https://waggawagga.motorprotein.de, ref. ^40^). The predicted interaction sites corresponded well with crystal structures of PPARγ in complex with agonists, coactivator peptides and its coiled-coil interaction partner RXRα. **d**–**f** Comparison of the modelled interactions of PEP (**d**), MP (**e**) and a SRC1-peptide (**f**, PDB code: 1fm9) with the coactivator interaction site. Cartoon representation of PPARγ is coloured in white, helices are numbered in black and the residues interacting with the peptides are shown as sticks in green and labelled in white with green contour. Leucines of the LXXXL motif of PEP and of the LXXLL motif of SRC1 and MP are shown as yellow sticks and labelled in yellow, SRC1 and the peptides are represented as blue cartoon. For the PEP and MP peptides, interactions were calculated with AMBER18^67^ from a 120 ns MD simulation. For SRC1, interactions were calculated from the 1fm9 structure. In both cases, the maximal interatomic contact distance was set to 5 Å, excluding hydrogens, and interactions were required to be present in more than 80% of the 50,000 frames analysed. The structure in the last frame of each simulation is shown.

We then used three different web servers to identify putative peptide binding sites on the 12-helices bundle of the ligand-binding domain (LBD) of PPARγ (Fig. 6a). This analysis yielded three candidate regions for interaction (Fig. 6b). The web server meta-PPISP^37^ predicted the residues surrounding the agonist-binding pocket of PPARγ to be the most likely interaction site on the protein^38^ (Fig. 6b), mainly formed by residues of helices 2ʹ and 3 and the loop between them^39^. The coiled-coil (CC) predictors, Waggawagga^40^ and DeepCoil^41^, predicted only helix 10/11 as a possible site for coiled-coil interaction on PPARγ. Helix 10 mediates heterodimerization between PPARγ and RXRα and forms a coiled-coil with helix 10 of RXRα (Fig. 6c). The PINTS web server^42^ yielded several crystal structures of PPARγ and related proteins with LXXLL motif-harbouring coactivators or corepressors^43–46^. The latter had been co-crystallized in an α-helical conformation at the coactivator interaction site^39^ which is formed by helices 3, 4, 5 and 12 of PPARγ (Fig. 6b).

As the heterodimerization and the coactivator-binding sites were already experimentally shown to be binding sites for α-helical peptides and protein regions, they were thought to be more likely interaction sites than the agonist-binding site. Docking was performed using two different web servers that employ different approaches: The web server GalaxyPepDock^47^ docked the MP exclusively with the canonical LXXLL motif at the coactivator binding site. The PEP was also docked exclusively to this site either with its LXXXL or LXXL motifs. The second tool used was the web server ClusPro^48^. The ClusPro docking resulted in complexes with the peptides docked to varying sites on PPARγ, including helix 10/11 and the proximity of the coactivator interaction site.

To assess which of these complexes might be the most favourable ones, all-atom molecular dynamics (MD) simulations followed by calculations of binding free energies were performed on 23 complexes from the two web servers with MP and PEP bound to different sites on the nuclear receptor. Peptides were bound either to the heterodimerization site or the coactivator interaction site. The results of the free energy calculations indicated that the coactivator interaction site is a more favourable binding site than the heterodimerization site (Tab. S3). Interactions were calculated for the most energetically favourable complex between each of the peptides and PPARγ, as well as for PPARγ with the classical coactivator peptide from SRC1. As seen in the last frame of the MD simulation, PEP bound to the coactivator interaction site with its N-terminal LXXXL motif (Fig. 6d), while MP and SRC1 bound to the interaction site with their canonical LXXLL motifs, respectively (Fig. 6e, f). Comparing the calculated interactions to those described by Nolte et al.^39^, the binding mode of MP seemed to be similar to SRC1, involving residues on helices 3, 4 and 12 (Tab. S4), including K301 on helix 3 and E471 on helix 12 (Fig. 6e). These two residues have been described to form a charged clamp that is thought to position the coactivator motif on SRC1 in the correct orientation at the coactivator interaction site^39^.

PEP was not found to interact with residues on helix 12 in more than 80% of the 120 ns MD simulation and, therefore, is unlikely to make use of the charged clamp for positioning (Fig. 6d). The leucine at position five in the LXXLL/LXXXL motifs engaged in the majority of the interactions by inserting into a hydrophobic cleft between helices 3, 4 and 5, as described for SRC1^39^. For both peptides, this leucine was calculated to interact with F306 inside this cleft, an interaction residue not shown for SRC1.

The structures of the complete complexes of PPARγ with PEP or MP at the last frame of the MD simulations are shown in Supplementary Fig. 4. Outside the coactivator interaction site, their conformations differ. PEP was found to drape its C-terminal end in a stable helix over the entrance of the agonist-binding pocket in proximity of helices 2ʹ and 3, while its N-terminus remained unstructured and did not form stable interactions. The N-terminus of MP stayed close to helices 7 and 11, while its C-terminus was close to helix 1.

In conclusion, the in silico prediction of the structures of peptide/PPARγ complexes indicates a coactivator-like binding mode that may explain the capacity of the peptides to stimulate the transcriptional activity of PPARγ.

## Discussion

In the present study, we describe a novel role for MTMR7 in human CRC, identifying it as a binding partner and positive regulator of PPARγ. The transcriptional activity of PPARγ is regulated by ligand binding, post-translational modifications and subcellular localisation^49^. The here described MTMR7-PPARγ interaction complex offers a novel mode of PPARγ regulation by compartmentalization and a possible explanation for the pro- vs. anti-tumour effects of PPARγ-agonists^50–52^. Previously, we characterized MTMR7 as an inhibitor of ERK1/2 and AKT/mTOR signalling^25^, thus acting as a dual blockage of two interconnected and compensatory oncogenic pathways downstream of receptor tyrosine kinases (e.g. the EGF receptor) and RAS^53,54^. MTMR7 may thus enforce the classical nuclear function of PPARγ by inhibiting the RAS-ERK1/2 cascade, which otherwise evokes inactivation of PPARγ by ERK1/2-mediated phosphorylation and MEK1/2-dependent sequestration of PPARγ in the cytosol^16^, where it interacts with molecules that restrain PPARγ activity including caveolin-1^26^ and heat shock proteins^55^.

In the current study, we provide evidence that MTMR7 also reduces the rapid “non-genomic activation” of ERK1/2 by the PPARγ-agonist rosi^56,57^. Through this mechanism, which has been shown to be mediated by EGF- or G-protein-coupled receptors (e.g. GPR40) in the plasma membrane, MTMR7 may prevent the adverse side effect of this class of PPARγ-ligands on cell proliferation and organ damage^49–52^. This finding is of particular significance in light of the plethora of negative clinical trials using PPARγ agonists in the therapy of gastrointestinal malignancies^14,58^: since the loss of MTMR7 is a common event in CRC, induced by stimuli including insulin or insulin-like growth factors^25^, the MTMR7 expression status might be a predictive parameter when targeting PPARγ in the setting of anti-proliferative therapies.

**Fig. 7:**
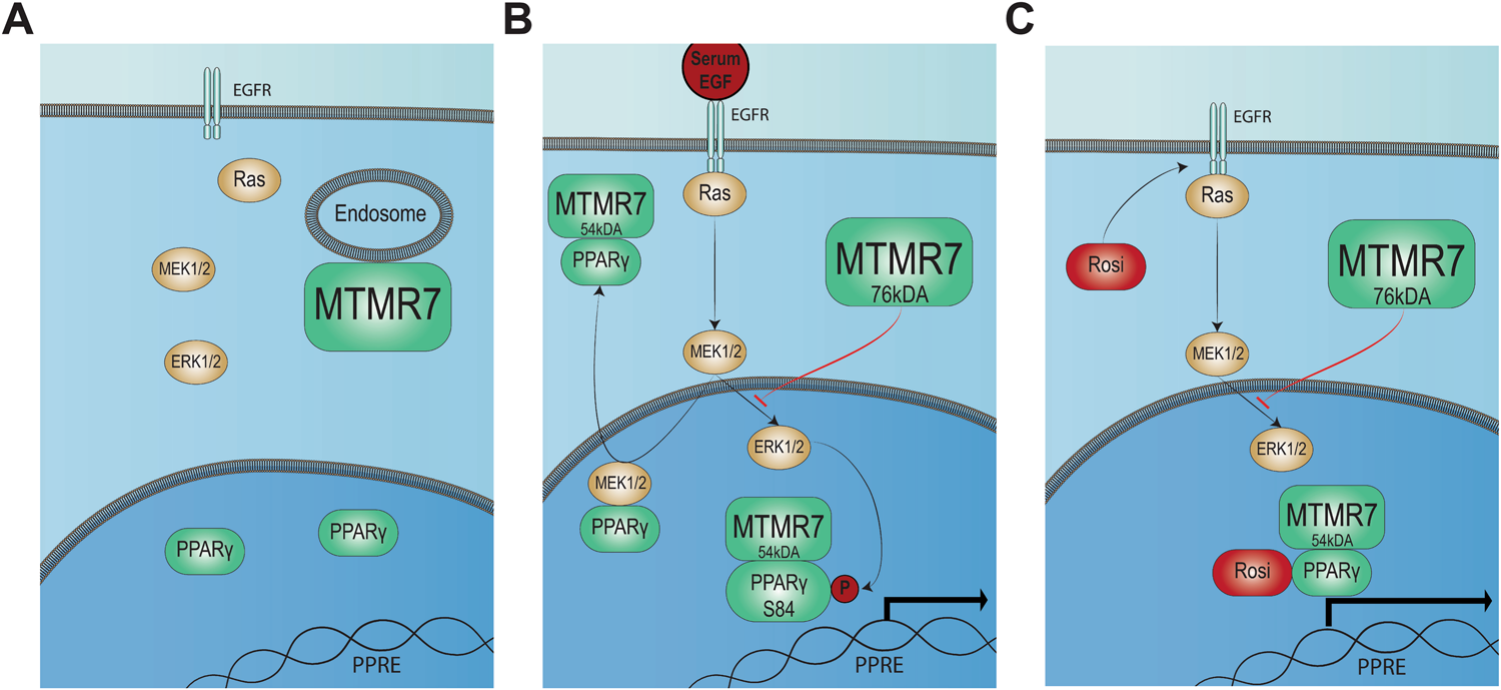
MTMR7-PPARγ signalling model. MTMR7 was identified as a novel PPARγ interactor, which increased PPARγ activity in vivo and in vitro. We propose a full-length (FL) isoform (76 kDa) in the cytosol, harbouring an N-terminal PH domain for binding to plasma and/or intracellular membranes (e.g. endosomes), and a mobile truncated isoform (54 kDa), presumably consisting of the C-terminal part of MTMR7 harbouring the coiled-coil (CC) domain, which was also found in the nucleus. **a** In resting cells, MTMR7 is bound to endosomes (or other vesicular structures), and ERK1/2 and MEK1/2 reside in the cytosol, whereas PPARγ is localized in the nucleus. **b** Upon stimulation with EGF (or serum), the kinases translocate into the nucleus where ERK1/2 phosphorylate PPARγ on Ser84, while MEK1/2 export PPARγ to the cytosol, two events resulting in inactivation of PPARγ. We suggest that MTMR7 counteracts the inhibitory effects of the two kinases: (1) MTMR7 FL stays in the cytosol and/or attached to membranes via its PH domain and inhibits MEK1/2-dependent ERK1/2 activation by an yet unknown mechanism, presumably involving altered PIP metabolism and EGFR traffic as shown for other MTMs. This event abrogates post-translational inactivation of PPARγ by the above mentioned kinases (in **b**), resulting in increased transcriptional activity of PPARγ. (2) Truncated MTMR7 translocates to the nucleus, directly binds to PPARγ via its coiled-coil (CC) domain and acts as a nuclear receptor coactivator harbouring LXXLL-like motifs similar to SRC1. Thereby, the transcriptional activity of PPARγ on target gene promoters is restored (e.g. *P21*^*CIP1/WAF1*^*,* e.a). **c** PPARγ-ligand (exemplified by rosi) binds to PPARγ and enhances its transcriptional activity in the nucleus. In addition, rosi triggers “non-genomic” trans-activation of plasma membrane receptors (e.g. EGFR, GPR40) and downstream kinases (in **b**). As for EGF/serum (in **b**), MTMR7 counteracts rosi-dependent “non-genomic” activation of MEKs/ERKs and promotes transcriptional activity of PPARγ in the nucleus together with its mode of action as a SRC1-like coactivator.

Moreover, we offer new insights into the subcellular distribution of MTMR7. While myotubularins in general are regarded to be cytosolic proteins^21,22^, we collected data suggesting that MTMR7 shuttles between the cytosol and the nucleus in response to stimuli of the RAS signalling cascade or to PPARγ agonist (here exemplary for rosi). In mice^24^, full-length (FL) 76 kDa and truncated 54 kDa isoforms of MTMR7 are expressed. The latter lacks the C-terminal coiled-coil (CC) domain and may thus be incapable of oligomerization with other MTMs^24^. The peptides identified in SW480 cells by MALDI-MS also suggest the existence of a 54 kDa isoform of MTMR7 in human CRC cells, forming the complex with PPARγ. Western blot analyses using four different Abs against the C-terminal domain of MTMR7, as well as previously published exon-selective RT-PCRs from several human CRC cell lines^25^ confirmed the presence of truncated <54 kDa MTMR7 (Supplementary Fig. 5). We may hence conclude that the identified isoform is truncated at the N-terminus, containing the PH domain, responsible for membrane association. This truncation might therefore enable MTMR7 to shuttle into the nucleus, together with its binding partner PPARγ (see model in Fig. 7), to enforce the transcriptional activity of the nuclear receptor.

This mode of action is further supported by the observed efficacy of the MTMR7-CC mimicry peptide, resembling the coiled-coil (CC) domain of the phosphatase, which was able to activate PPARγ in vitro and in vivo: Notably, it induced transcription of PPARγ target genes as well as body weight gain, a well-known adverse effect attributed to PPARγ agonists of the thiazolidinedione class (e.g. rosi)^49,59,60^.

Exploitation of different web servers for binding site prediction and docking, in combination with MD simulations, pointed at the coactivator interaction site of PPARγ being the most likely binding site for the peptides, similar to those of the bona fide coactivator SRC1, inserting the leucine at position 5 of its LXXLL motif into a hydrophobic cleft between helices 3, 4, 5 and 12^38^. Complexes covering this interaction site were more stable and energetically favourable compared with any other candidate sites like the heterodimerization interface or the agonist-binding pocket. MP and PEP interacted with residues that were also reported for SRC1 by Nolte et al.^39^. As such, MP made use of the charged clamp between E471 and K301 and interacted with several residues on helix 12. Both peptides were also found to interact with F306, implicating that the LXXLL/XXXL motifs might reach further into the cleft and contribute to the stable binding of the peptides to the coactivator interaction site. Notably, stretches of the peptides unfolded from their initial helical conformation assigned, whereas other parts, and especially the ones bound to the coactivator-binding site, stayed helical throughout the MD simulations. The stable helix formation of this peptide region agrees with reports on the behaviour of the LXXLL motif in SRC1 that is thought to be unstructured in its apo-form but to form a short amphiphatic α-helix upon binding to the coactivator-binding site^61,62^.

While the computational methods favour the coactivator interaction site and resulted in a seemingly plausible interaction prediction, one important short-coming of this model for the PPARγ/PEP complex is that PEP does not contain the canonical LXXLL motif that was deemed to be necessary for this kind of coactivator-like interaction in the past^62^. The fact that MP gave more favourable binding free energies than PEP supports the reported importance of all three leucines. Cell-free competition assays with both peptides and SRC1 on recombinant PPARγ-LBD protein would be necessary to experimentally confirm our in silico predictions.

Preliminary findings revealed that the peptides were unable to displace rosi from the PPARγ-LBD, thus unlikely to act as true agonists; instead, rosi increased MTMR7/PPARγ complex formation and MTMR7 protein expression in CRC cells (not shown). These observations may indicate that, alike MTMR9, ligand-activated PPARγ could stabilize the MTMR7 protein, and, vice versa, MTMR7 may lead to a quasi-allosteric activation of PPARγ via the SRC1 coactivator site. However, future in depth nuclear magnetic resonance (NMR) or co-crystallization studies will be required to experimentally prove the predicted in silico models.

Taken together, our study identified a novel positive regulator of PPARγ based on (i) inhibition of ERK1/2 signalling and (ii) direct interaction with the CC domain of MTMR7. Additional studies will be necessary to characterize the pharmacological properties of the peptides to allow further development of this novel PPARγ activator for future clinical applications.

## Materials and methods

### Reagents and plasmids

Chemicals were from Merck/Sigma (Darmstadt, Germany). Antibodies (Abs) used are listed in Table. S2. Untagged and GFP-tagged PPARγ1 and PPRE-luc plasmids were mentioned previously^15^. Human full-length (FL) MTMR7 cDNA (start codon MEHIRT, aa 1–660, 76 kDa, NM_004686.4) was in pTarget (pT) vector (Promega GmbH, Mannheim, Germany) with or without GFP-tag^25^. Transient transfection and luciferase assays were performed as described^63^.

### Peptides

PEP corresponded to the original leucine-rich aa sequence of MTMR7 (aa 521–550; SwissProt ID: Q9Y216.3: **L**MAVKEETQQ**L**EEE**L**EA**L**EER**L**EKIQKVQ**L**) (Tab. S1). MP was designed based on the PEP sequence using a random scrambling web tool (https://web.expasy.org/randseq/: VLQEEILEMTEEKA*LLQALL*KKEQERVQEE) generating a canonical *LXXLL* nuclear receptor coactivator motif. Both peptides were synthesized by automated, solid phase peptide synthesis on Rink amide resin and coupled at the N-terminus with myristidic acid (ETH Zürich, Switzerland). The resulting peptides were characterised by HPLC and MALDI-MS for purity and sequence confirmation and provided as lyophilized powders^64^.

### Cell culture

Human embryonic kidney (HEK293T) and colon adenocarcinoma cell lines (all from the American Type Culture Collection, Rockville, MD) were maintained as before^26^. PDOs from CRC tissues were cultivated as published in refs. ^65,66^. All cultures were routinely tested for contamination with Mycoplasma (InVivogen, Toulouse, France).

## Supplementary information

supplement methods R2

supplement figure legends R2

supplement figure S1

supplement figure S2

supplement figure S3

supplement figure S4

supplement figure S5

supplement tables R2
