# Supplementary material for: Myotubularin-related protein 7 activates peroxisome proliferator-activated receptor-gamma
Source: Oncogenesis. 2020 Jun 10;9(6):59. doi: 10.1038/s41389-020-0238-8 (PMC7286916; doi:10.1038/s41389-020-0238-8)
Supplement: Supplementary file 1 — supplement methods R2 [file 41389_2020_238_MOESM1_ESM.docx]

**Supplementary** **Materials and Methods**

**Patients**

Tissue microarrays (TMAs) of CRC patients (n=48 cases) were purchased from US Biomax (Co483, Rockville, MD) or were kindly provided by C. Röcken (Pathology, Kiel) (1). Patient studies were conducted in accordance with ethical guidelines (Declaration of Helsinki). All patients provided written informed consent for the analysis of molecular parameters. Specimens were collected by surgery resection or as endoscopic biopsy prior to any therapy and subjected to histological evaluation according to the Vienna classification (2). Generation of and experiments with patient-derived organoids (PDOs) (3, 4) were approved by the Medical Ethics Committee II of the Medical Faculty Mannheim, Heidelberg University (2014-633N-MA; 2016-607N-MA).

**Animals**

C57BL/6J [CEA424-SV40 Tag] mice were described before (5). Animal studies were conducted in agreement with ethical guidelines of the University of Heidelberg and approved by the government of Baden-Württemberg, Karlsruhe (G-188/18). Sample sizes of n≤10 per group were chosen. Female animals were randomized according to age (2 months) and body weight (20 g) and received food and water *ad libitum* before drug administration. Peptides were dissolved in sterile DMSO diluted in PBS and administered i.p. as a single dose of 30 mg/kg per day, 4 times per week for 2 weeks. Thereafter, animals were sacrificed and organs snap-frozen in liquid N_2_ or processed for formalin-fixation and paraffin-embedding (FFPE). Analyses were conducted observer-blinded.

**Subcellular fractionation (SCF), MALDI-MS, co-immunoprecipitation (CoIP), Western blot**

Methods were performed as detailed elsewhere (6, 7). Briefly, cytosolic lysates were prepared from cells upon detergent-free hypotonic lysis and subjected to immunoprecipitation (IP), followed by SDS-PAGE and detection of the precipitated proteins by Western (immuno) blot (IB). For MALDI-MS, immunoprecipitates were separated by SDS-PAGE and detected by silver staining as detailed previously (6). Bands were cut from gels and sequenced using GPS Explorer 2 software (Applied Biosystems/Thermofisher Scientific) in cooperation with the Dept. of Proteomics and Bioanalytics (Technische Universität München, Germany) (8). Subcellular fractionation (SCF) followed a previous protocol (6, 7).

**Proximity ligation assay (PLA) and immunofluorescence**

Ab stainings and image acquisition were conducted as described (9, 10) and as recommended by the manufacturer (Duolink®, Merck).

**Reverse transcription PCR (RT-PCR) and quantitative PCR (qPCR)**

These methods were performed as published (11, 12) and oligonucleotides are listed in **Tab.S2**.

**Immunohistochemistry (IHC)**

All stainings were done as described (13). In brief, antigen retrieval was performed on deparaffinized tissue or PDO sections using citrate buffer and incubated with H_2_O_2_ to block endogenous peroxidase activity. IHC was then done using Abs (at a dilution of 1:100-1:200), followed by detection with 3,3'-diamino benzidine (VectorLabs, Peterborough, UK) (brown color) and counterstaining with haematoxylin (blue color). Nuclear and cytoplasmic staining positivity was counted in epithelial (tumor, TU; normal colon, NC) and stroma (lamina propria) cells. Staining frequency and intensity was evinced in commercial TMAs (Co483, US Biomax, Rockville, MD): Scores: 0+ = negative (0-25% positive rate), 1+ = weak (25-50%), 2+ = moderate (50-75%), 3+ = strong (75-100%). In addition, dichotome distribution of staining positivity was applied (0 = negative *vs*. 1 = positive). The analyses were conducted observer-blinded using standard bright-field microscopy and manual counting supported by Image J (imagej.nih.gov/ij).

**Statistics and software**

Sample sizes were chosen after POWER analysis using SAS (v.9.3, SAS Institute, Cary, NC) with Nominal Alpha 0.05 and Nominal Power 0.8. Results are expressed as means ± S.E. from at least 3 independent, repeated experiments from (i) the same commercial cell line or PDO line from different, consecutive cultivation passages, herewith defined in the text as “replicates”, or (ii) from different individuals, termed patients’ cases or mice. Optical density (O.D.) values from bands in gels of Western blots were normalized to those from house keeper proteins (β-tubulin, HSP90, Lamin A/C) and calculated as -fold of control as indicated in the legends to figures. Ct values from RT-qPCRs were normalized to those of the respective house keeper gene (*B2m)* and calculated according to the ΔΔCt method as recommended in (14). Statistical analysis was performed using Graphpad Prism (version 6.0, La Jolla, CA). For each data set, distribution of normality and estimates of variation were calculated before assignment to an appropriate test. All tests were two-sided, and p-values <0.05 were considered significant and marked by asterisk (*). Open access web tools were RCSB Protein Data Bank (PDB), NCBI (BLASTp) and cBioPortal of Cancer Genomics (15).

**Computational Methods** - **Prediction of potential peptide binding sites on PPARγ**

Coordinates of the structures of human PPARγ (PDB ID: 1fm6, 2.1 Å resolution, 1fm9, 2.1 Å resolution (16)) were retrieved from the RCSB-Protein Data Bank (17). Water and other co-crystallized molecules were removed. PPARγ structures used corresponded to chain X from 1fm6 and chain D from 1fm9. These PPARγ structures were submitted in pdb format to the metaPPISP web server (http://pipe.scs.fsu.edu/meta-ppisp.html, (18)) with the threshold set to 0.34 and to the PINTS web server (http://www.russelllab.org/cgi-bin/tools/pints.pl, (19)) for prediction of possible interaction sites. E-values of ≤ 10 were implemented and the PINTS web server was set to compare the inserted protein against structural patterns and to give less than 100 hits.

For specific prediction of potential coiled-coil interaction sites, the amino acid sequence of the PPARγ structures from the pdb files was submitted to the DeepCoil web server (https://toolkit.tuebingen.mpg.de/#/tools/ deepcoil, (20)) and to the Waggawagga web server (https://waggawagga.motorprotein.de/,(21)). On Waggawagga, all prediction tools were selected and the window length was set to 21 residues.

**Modeling of PEP and MP**

The secondary structure of the peptides was predicted by submitting their amino acid sequences to the web servers PredictProtein (https://predictprotein.org/, (22)) and Agadir (http://agadir.crg.es/, (23)). The Agadir settings were: pH 7, temperature 300 K, ionic strength 0.1 M and no capping of the termini. Monomeric peptides were built with ProBuilder (https://nova.disfarm.unimi.it/ probuilder.htm) with the settings: Secondary structure type alpha helix, phi -135 °, psi 135 °, omega 180 ° and addition of the side chains. The peptides were protonated using PDB2PQR (http://nbcr-222.ucsd.edu/pdb2pqr_2.0.0/, (24)), and energy was optimized using MOE 2019.1, with the Amber10 force field and a root mean square (RMS) gradient of 0.1 kcal/mol/Å^2^ as the convergence criterion (Molecular Operating Environment 2019).

**Docking of PEP and MP to PPARγ**

PPARγ and the monomeric peptides, both protonated by PDB2PQR, were submitted in pdb format to the web server ClusPro (https://cluspro.bu.edu/, (25)). With a length of 30 aa, the peptides were too long to be docked with the peptide docking tool of ClusPro and were therefore docked like rigid helical proteins. PPARγ was submitted in pdb format to GalaxyPepDock, without hydrogens, as submitting the protonated protein led to an error in the docking trial (http://galaxy.seoklab.org/ cgibin/submit.cgi?type=PEPDOCK, (26)). In addition, the amino acid sequences of the peptides were submitted to Galaxy PepDock.

**All-atom molecular dynamics (MD) simulation**

A total of 23 peptide/PPARγ complexes were selected for MD simulation: 17 of these were from GalaxyPepDock: PEP docked to the PPARγ from 1fm9(#4) and from 1fm6(#5), MP docked to the PPARγ from 1fm9 (#4) and 1fm6 (#4). Six complexes were ClusPro docking solutions: PEP docked to PPARγ from 1fm6 (#1) and from 1fm9 (#2), and MP docked to PPARγ from 1fm6 (#3). The structures were prepared for all-atom MD simulation using the tleap program in the AMBER MD package version 18 (http://ambermd.org/, (27)). The AMBER ff14SB forcefield was used to assign parameters to the complexes (28). The systems were solvated in a periodic box using the “transferable intermolecular potential with 3 points” (TIP3P) water model (29) and neutralized using 11 Na+ ions and 1 Cl- ion. The distance from the edge of the rectangular box to the solute was 10 Å. MD simulation was performed with a 10 Å non-bonded cut-off, long range interactions were treated using the Particle Mesh Ewald (PME) method. During energy minimization, no bond lengths were constrained, whereas from the heating step onwards bonds to hydrogen atoms were constrained. Each system was energy minimized three times with 500 steps of steepest descent followed by 1000 steps of conjugate gradient minimization (first with a convergence criterion of 5 kcal/mol-Å, then two times with a convergence criterion of 0.001 kcal/mol-Å) and one time with 100 steps of steepest descent followed by 1400 steps of conjugate gradient minimization (with a convergence criterion of 0.001 kcal/mol-Å). For heating, the Langevin thermostat (NVT ensemble) was used with a collision frequency of 1 per ps. The initial temperature was set to 10 K and the system was heated to 300 K in 990 ps using a timestep of 2 fs. An initial equilibration was performed for 1 ns using a timestep of 1 fs under isothermic-isobaric conditions using the default Berendsen barostat with isotropic position scaling. The reference pressure was 1 bar, and the pressure relaxation time was 1 ps.

Then, a second equilibration step was performed under isothermic-isobaric conditions with the default Berendsen barostat for 20 ns with a timestep of 2 fs. Finally, a production run was performed with a timestep of 2 fs for 100 ns. The final complete trajectory had 50.000 frames with frames recorded every 2 ps. The MD trajectories were analysed using VMD (https://www.ks.uiuc.edu/Research/vmd/, (30)) and visualization was done with VMD (version 1.9.2) and Pymol (version 1.8.2.3) (https://pymol.org/).

**Calculation of binding free energies**

To calculate the relative free energies of binding using Generalized Born Surface Area calculations, the MMPBSA.py script (31) was run in AMBER. Calculations were performed excluding the first 12.500 frames from each trajectory, which corresponded to the equilibration of the system. Structures were collected every 0.2 ns.

**Interaction analysis**

Interactions between PPARγ and PEP or MP were analyzed using the CPPTRAJ module of AMBER18 (32) using the whole trajectory from the MD simulation. An interaction was considered to be present when an atom from one molecule was within 5 Å of an atom of the other molecule and stayed in this proximity for 80 % of the 50.000 frames of the simulation. Visualization was performed with Pymol. Interactions between PPARγ and SRC1 were calculated under the same proximity conditions from the 1fm9 crystal structure (chains D and E).

**References**

1. Friedrich T, *et al.* Subcellular compartmentalization of docking protein-1 contributes to progression in colorectal cancer. *EBioMedicine* 2016; **8:** 159-172.

2. Schlemper RJ, *et al.* The Vienna classification of gastrointestinal epithelial neoplasia. *Gut* 2000; **47:** 251-255.

3. Zhan T, *et al.* MEK inhibitors activate Wnt signalling and induce stem cell plasticity in colorectal cancer. *Nature Communications* 2019; **10:** 2197-2213.

4. Betge J, *et al.* Multiparametric phenotyping of compound effects on patient derived organoids (doi.org/10.1101/660993). *bioRxiv,* Cold Spring Harbour Laboratory, USA, 2019.

5. Thompson J, *et al.* A transgenic mouse line that develops early-onset invasive gastric carcinoma provides a model for carcinoembryonic antigen-targeted tumor therapy. *Int J Cancer* 2000; **86:** 863-869.

6. Burgermeister E, *et al.* The Ras inhibitors caveolin-1 and docking protein 1 activate peroxisome proliferator-activated receptor gamma through spatial relocalization at helix 7 of its ligand-binding domain. *Mol Cell Biol* 2011; **31:** 3497-3510.

7. Burgermeister E, *et al.* Interaction with MEK causes nuclear export and downregulation of peroxisome proliferator-activated receptor gamma. *Mol Cell Biol* 2007; **27:** 803-817.

8. Wilhelm M, *et al.* Mass-spectrometry-based draft of the human proteome. *Nature* 2014; **509:** 582-587.

9. Ebert MP, *et al.* TFAP2E-DKK4 and chemoresistance in colorectal cancer. *N Engl J Med* 2012; **366:** 44-53.

10. Burgermeister E, *et al.* Differential expression and function of caveolin-1 in human gastric cancer progression. *Cancer Res* 2007; **67:** 8519-8526.

11. Xing X, *et al.* Hematopoietically expressed homeobox is a target gene of farnesoid X receptor in chenodeoxycholic acid-induced liver hypertrophy. *Hepatology* 2009; **49:** 979-988.

12. Regel I, *et al.* Pan-histone deacetylase inhibitor panobinostat sensitizes gastric cancer cells to anthracyclines via induction of CITED2. *Gastroenterology* 2012; **143:** 99-109 e10.

13. Weidner P, *et al.* Myotubularin-related protein 7 inhibits insulin signaling in colorectal cancer. *Oncotarget* 2016; **7:** 50490-50506.

14. Pfaffl MW. A new mathematical model for relative quantification in real-time RT-PCR. *Nucleic Acids Research* 2001; **29:** e45-e45.

15. Cerami E, *et al.* The cBio cancer genomics portal: an open platform for exploring multidimensional cancer genomics data. *Cancer Discov* 2012; **2:** 401-404.

16. Gampe RT, Jr., *et al.* Asymmetry in the PPARgamma/RXRalpha crystal structure reveals the molecular basis of heterodimerization among nuclear receptors. *Mol Cell* 2000; **5:** 545-555.

17. Berman HM, *et al.* The Protein Data Bank. *Acta Crystallogr D Biol Crystallogr* 2002; **58:** 899-907.

18. Qin S, Zhou HX. meta-PPISP: a meta web server for protein-protein interaction site prediction. *Bioinformatics* 2007; **23:** 3386-3387.

19. Stark A, Sunyaev S, Russell RB, Russell RB. A model for statistical significance of local similarities in structure. *Journal of Molecular Biology* 2003; **326:** 1307-1316.

20. Ludwiczak J, Winski A, Szczepaniak K, Alva V, Dunin-Horkawicz S. DeepCoil - a fast and accurate prediction of coiled-coil domains in protein sequences. *Bioinformatics* 2019; **35:** 2790-2795.

21. Simm D, Hatje K, Kollmar M. Waggawagga: comparative visualization of coiled-coil predictions and detection of stable single alpha-helices (SAH domains). *Bioinformatics* 2015; **31:** 767-769.

22. Rost B, Yachdav G, Liu J. The PredictProtein server. *Nucleic Acids Res* 2004; **32:** W321-326.

23. Muñoz V, Serrano L. Development of the multiple sequence approximation within the AGADIR model of α-helix formation: Comparison with Zimm-Bragg and Lifson-Roig formalisms. *Biopolymers* 1997; **41:** 495-509.

24. Dolinsky TJ, Nielsen JE, McCammon JA, Baker NA. PDB2PQR: an automated pipeline for the setup of Poisson-Boltzmann electrostatics calculations. *Nucleic Acids Res* 2004; **32:** W665-667.

25. Kozakov D, *et al.* The ClusPro web server for protein-protein docking. *Nat Protoc* 2017; **12:** 255-278.

26. Lee H, Heo L, Lee MS, Seok C. GalaxyPepDock: a protein-peptide docking tool based on interaction similarity and energy optimization. *Nucleic Acids Res* 2015; **43:** W431-435.

27. Case DA, *et al. AMBER 2018*, University of California, San Francisco, USA, 2018.

28. Maier JA, *et al.* ff14SB: Improving the Accuracy of Protein Side Chain and Backbone Parameters from ff99SB. *Journal of Chemical Theory and Computation* 2015; **11:** 3696-3713.

29. Price DJ, III CLB. A modified TIP3P water potential for simulation with Ewald summation. *The Journal of Chemical Physics* 2004; **121:** 10096-10103.

30. Humphrey W, Dalke A, Schulten K. VMD: Visual molecular dynamics. Journal of *Molecular Graphics* 1996; **14:** 33-38.

31. Miller BR, 3rd, *et al.* MMPBSA.py: An Efficient Program for End-State Free Energy Calculations. *J Chem Theory Comput* 2012; **8:** 3314-3321.

32. Roe DR, Cheatham TE, 3rd. PTRAJ and CPPTRAJ: Software for Processing and Analysis of Molecular Dynamics Trajectory Data. *J Chem Theory Comput* 2013; **9:** 3084-3095.
