# Supplementary material for: Myotubularin-related protein 7 activates peroxisome proliferator-activated receptor-gamma
Source: Oncogenesis. 2020 Jun 10;9(6):59. doi: 10.1038/s41389-020-0238-8 (PMC7286916; doi:10.1038/s41389-020-0238-8)
Supplement: Supplementary file 2 — supplement figure legends R2 [file 41389_2020_238_MOESM2_ESM.docx]

**Legends to Supplementary Figures**

**S1 Supportive data on MTMR7**

**A,** MTMR7 reduces PPARγ-agonist-mediated activation of ERK1/2. SW480 cells were transfected with MTMR7 FL plasmid for 6 h, followed by serum-deprival for 16 h and subsequent stimulation with rosi (10 µM). IB of total cell lysates showed reduced ERK1/2 phosphorylation in presence of MTMR7. Data are absolute O.D. values normalized to HSP90 ± S.E. (t=30 min: p=0.0625 EV *vs*. MTMR7; Wilcoxon Signed Rank test, n=3 replicates).

**B-C,** *In silico* analysis of MTMR7-CC mimicry peptide. B, Coiled coil-forming capacity of MTMR7. The sequence started at amino acid (aa) position 11, N-terminal to the first aa of the peptide, and was aligned to the first lysine (K) of MTMR2 required for heterodimerization. NCOILS version 1.0 (ExPASy) was used to predict coil forming capacity of a 45 aa region aligning to MTMR2. A 30 aa region with a high capacity for coil formation was selected. On the x-axis, the 45 aa are plotted against the likelihood for coiled-coil formation on the y-axis. C, Using pepwheel provided by EMBOSS, the selected peptide was predicted to form an amphipathic helix. Color legend: red diamonds = neg. charged/hydrophilic aa; blue squares = hydrophobic aa; black circles = pos. charged/basic aa.

**S2 Results of secondary structure prediction by PredictProtein (https://predictprotein.org/)**

AA represents the amino acid sequence of the peptides with neutral residues in black, acidic residues in red and basic residues in blue. OBS_sec indicates numbers for experimentally observed secondary structures. PROF_sec shows a red H below the residues that it predicts to be part of a helix. Rel_sec gives a value of reliability for the secondary structure prediction of each residue with 9 being very reliable and 0 being not reliable. Sub_sec gives a subset of predictions based on the reliability. It only predicts structures for residues with a reliability ≥5 which correspond to a predicted accuracy of 82 % and also includes loops (L) in green.

**S3 Results of secondary structure prediction by Agadir (http://agadir.crg.es/)**

Data are giving the probability of a residue to be part of a Helix (Hel) in percent as well as the N-terminal and C-terminal residues of the helical state (Ncap and Ccap). **A,** Results for PEP. **B,** Result for MP. **C,** Results for PEP projected to the cartoon representation of the peptide. The MTMR7-CC peptide (PEP) is shown in the conformation it has in the last frame of the MD simulation bound to the co-activator interaction site of PPARγ. **D,** Results for the modified peptide (MP) projected to the cartoon representation of the peptide. The peptide is shown in the conformation it has in the end of the last frame of the MD simulation bound to the co-activator interaction site of PPARγ.

**S4 Cartoon of the peptide/protein complex in the last frame of the 120 ns MD simulation**

**A,** PEP/PPARγ-complex with the LXXXL motif of the peptide at the co-activator interaction site. **B,** MP/PPARγ-complex with the LXXLL motif of the peptide at the co-activator interaction site.

**S5 Detection of endogenous MTMR7 protein variants**

Human CRC and HEK293T (non-cancer) cell lines were transfected with EV or MTMR7 FL plasmid (lane 1 = positive control) for 48 h. Total cell lysates were subjected to IB using an Ab (#150458) specific for the C-terminus of the MTMR7 protein. Note that bands were detected corresponding to the 76 kDa MTMR7 FL protein, with highest levels in HCT116 cells, and a smaller band of ≤54 kDa in all cell lines tested.
