# Supplementary figures and images for: Myotubularin-related protein 7 activates peroxisome proliferator-activated receptor-gamma
Source: Oncogenesis. 2020 Jun 10;9(6):59. doi: 10.1038/s41389-020-0238-8 (PMC7286916; doi:10.1038/s41389-020-0238-8)

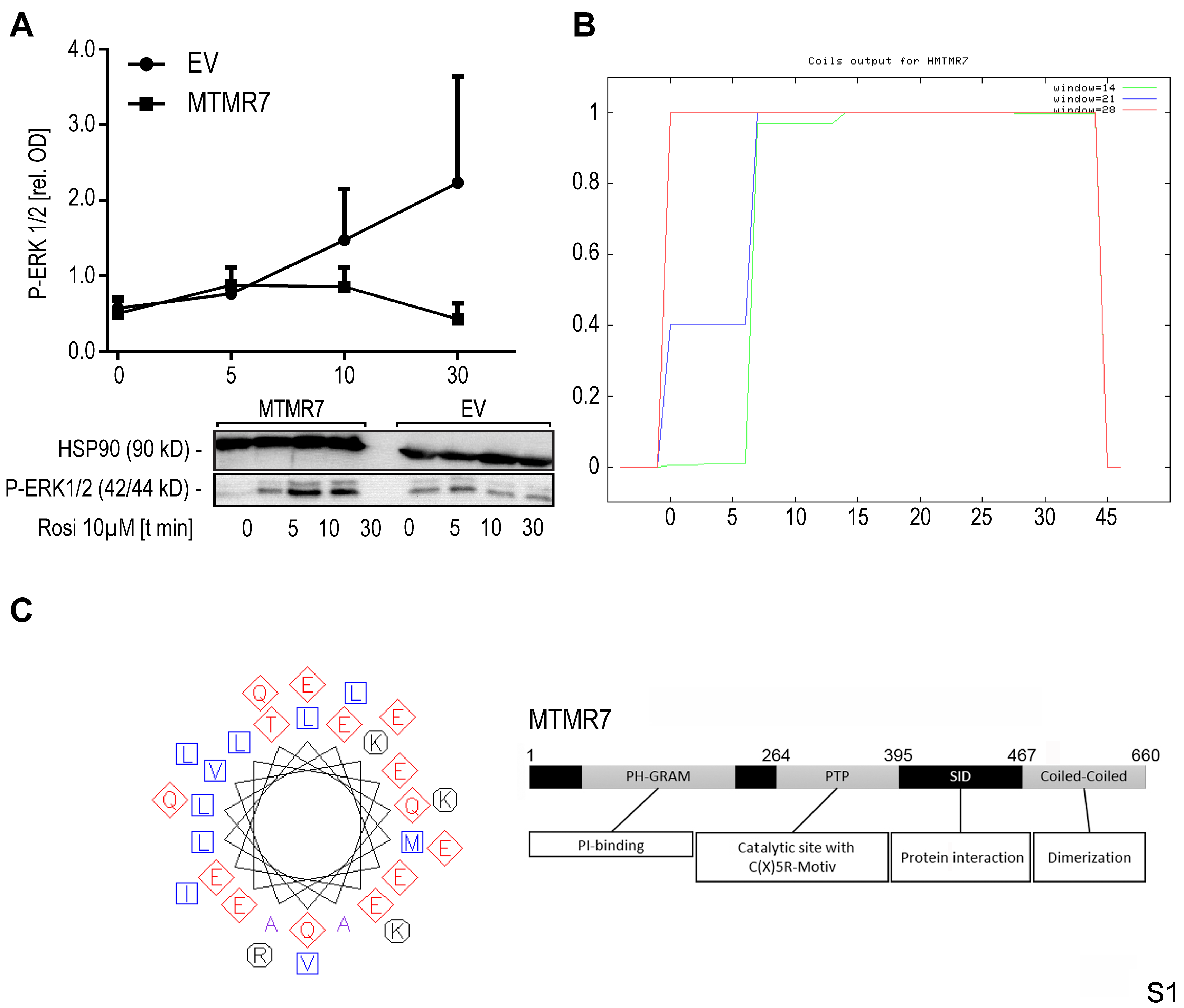

Supplement: Supplementary file 3 — supplement figure S1 [file 41389_2020_238_MOESM3_ESM.tif]

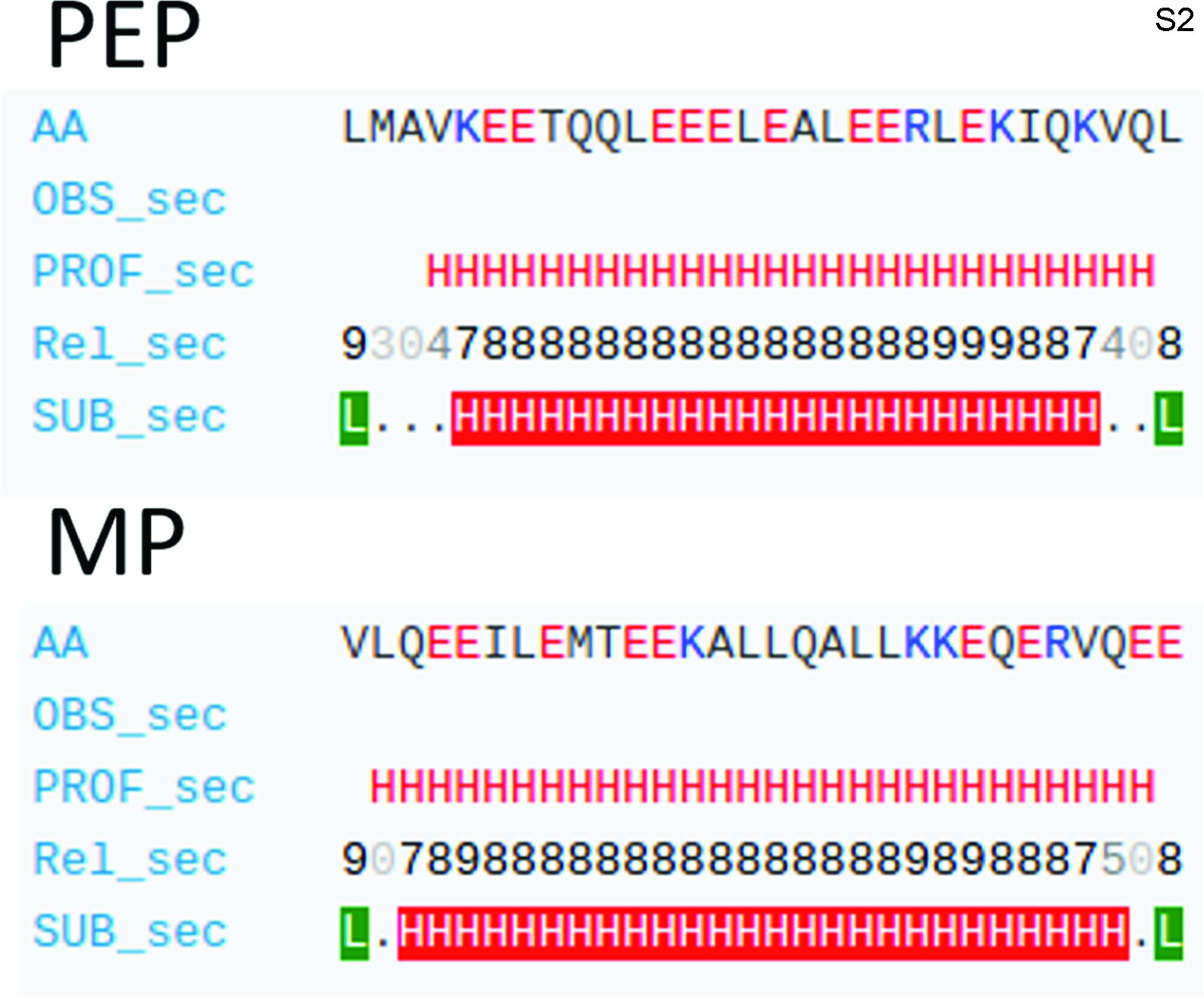

Supplement: Supplementary file 4 — supplement figure S2 [file 41389_2020_238_MOESM4_ESM.tif]

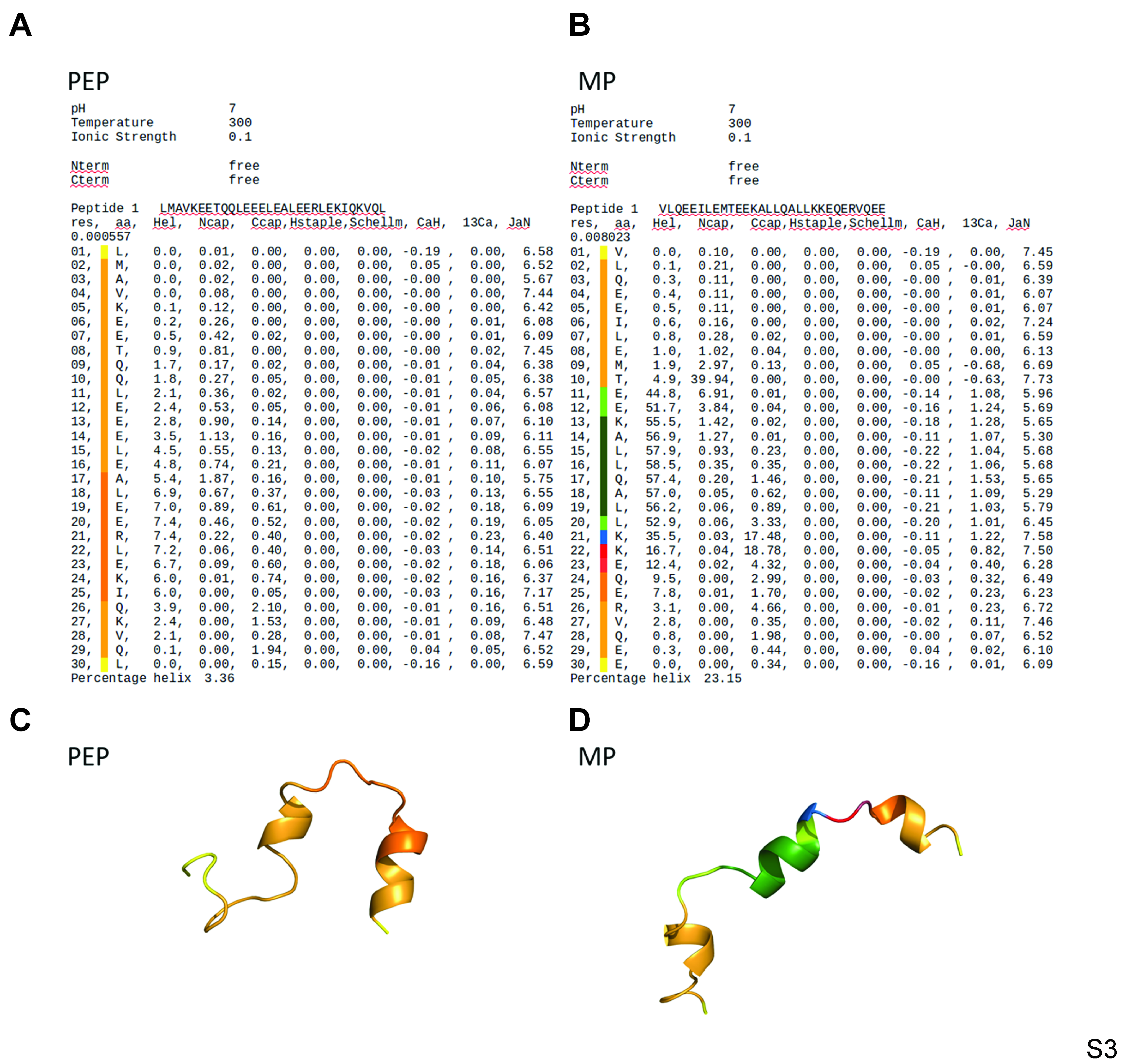

Supplement: Supplementary file 5 — supplement figure S3 [file 41389_2020_238_MOESM5_ESM.tif]

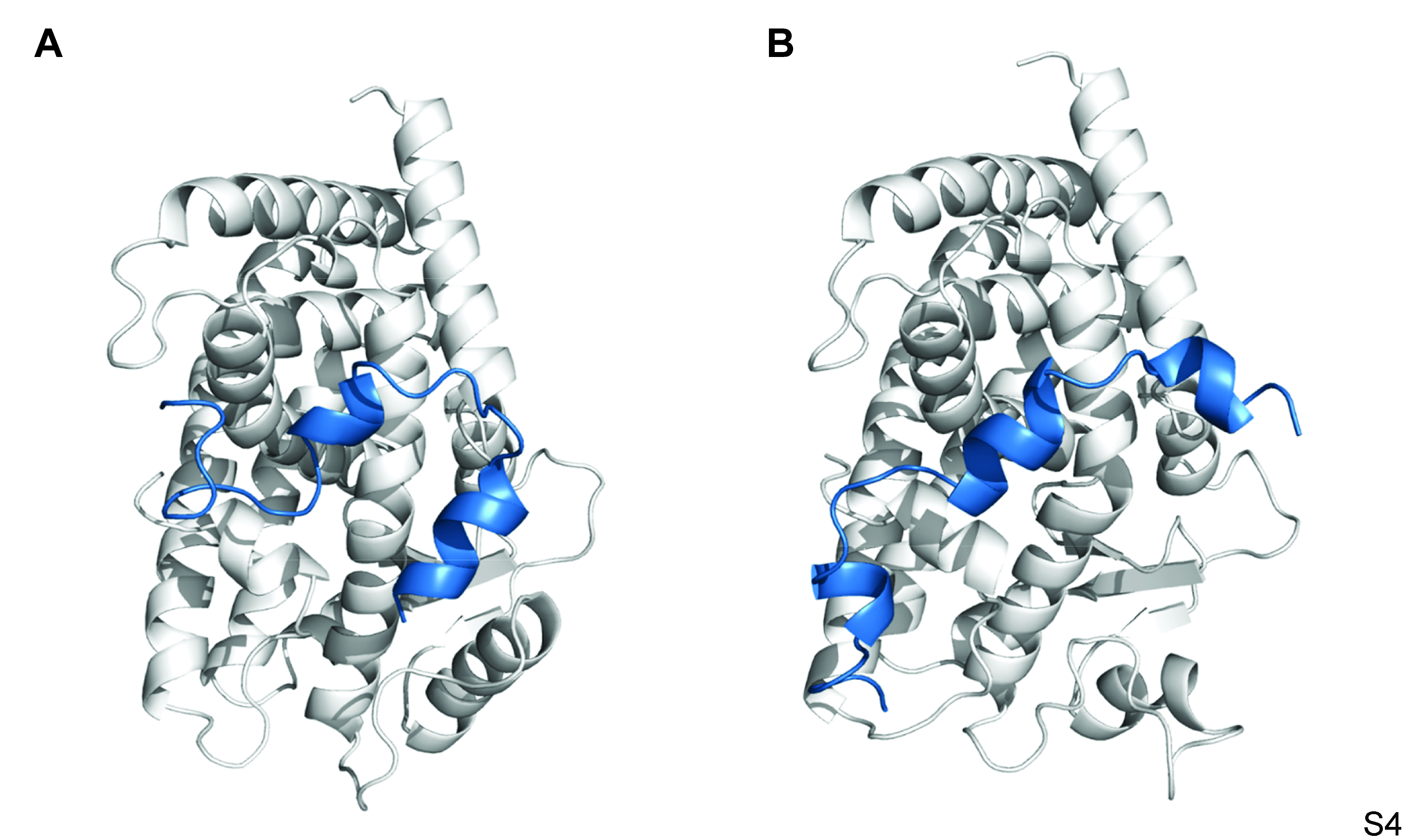

Supplement: Supplementary file 6 — supplement figure S4 [file 41389_2020_238_MOESM6_ESM.tif]

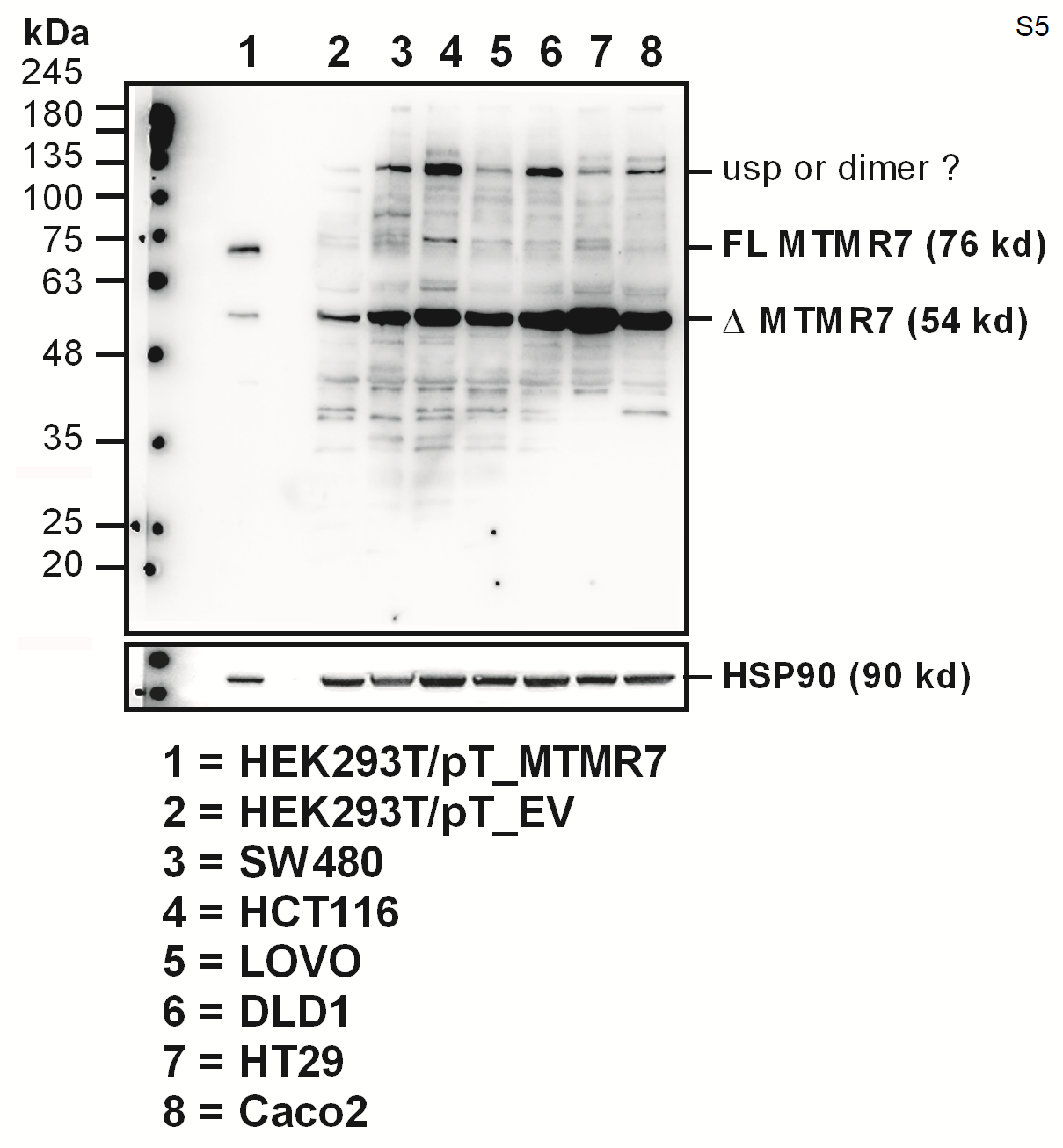

Supplement: Supplementary file 7 — supplement figure S5 [file 41389_2020_238_MOESM7_ESM.tif]
