# Supplementary material for: Myotubularin-related protein 7 activates peroxisome proliferator-activated receptor-gamma
Source: Oncogenesis. 2020 Jun 10;9(6):59. doi: 10.1038/s41389-020-0238-8 (PMC7286916; doi:10.1038/s41389-020-0238-8)
Supplement: Supplementary file 8 — supplement tables R2 [file 41389_2020_238_MOESM8_ESM.doc]

**Supplementary Tables**

| **Table S1 MTMR7 peptides ^#^ identified by MALDI-MS ***  ***** Mascot total ion score by GPS Explorer 2 software  (Applied Biosystems/Thermofisher Scientific). | | | | | |
| --- | --- | --- | --- | --- | --- |
| **Accession** | **Mass (Dalton)** | **Score** | **Description** | | **Species** |
| Q9Y216 | 76.754 | 79 | **Myotubularin-related protein 7** | | HUMAN |
| **^#^** Location of peptides identified by MALDI-MS in the coding sequence (CDS)  of human MTMR7 protein aligned with BlastP software (NCBI). | | | | | |
| **Mass (Dalton)** | **CDS location** | | | **Peptide** | |
| 1.009,5110  1.011,4821  1.090,5576  1.099,5962  1.109,5675  1.109,5675  1.179,6238  1.234,6943  1.254,6118  1.378,6467  1.481,7448  1.481,7448  1.543,7516  1.543,7516  1.621,8342  1.621,8342  1.634,9337  1.634,9337  1.650,9259  2.264,1860 | CC domain (C-terminus)  PH-GRAM domain (N-terminus)  CC domain (C-terminus)  PH-GRAM domain (N-terminus)  SET and CC domains (C-terminal)  SET and CC domains (C-terminal)  Catalytic phosphatase domain  CC domain (C-terminus)  SET and CC domains (C-terminal)  SET and CC domains (C-terminal)  SET and CC domains (C-terminal)  SET and CC domains (C-terminal)  Catalytic phosphatase domain  Catalytic phosphatase domain  Catalytic phosphatase domain  Catalytic phosphatase domain  Catalytic phosphatase domain  Catalytic phosphatase domain  Catalytic phosphatase domain  Catalytic phosphatase domain | | | R.FEKGMQPR.Q  -.MEHIRTPK.V  K.VQLNCTKVK.S  K.VENVRLVDR.V  R.ADYLNPLFR.A  R.ADYLNPLFR.A  R.FPVLSYYYK.D  K.IQKVQLNCTK.V  R.QSVTDYLMAVK.E  K.NRADYLNPLFR.A  K.FWSGMYNRFEK.G  K.FWSGMYNRFEK.G  R.HIKAIMDAGIFIAK.A  K.DWISFGHKFNHR.Y  K.FQFIGIENIHVMR.N  K.FQFIGIENIHVMR.N  R.CLEDEQMLQAIRK.A  R.CLEDEQMLQAIRK.A  R.CLEDEQMLQAIRK.A  R.MGLPNHYWQLSDVNRDYR.V | |

| **Table S2 Primers * and Antibodies #**  * Primers were used for RT-qPCR; # Antibodies for immunohistochemistry (IHC), immunofluorescence microscopy (IF), immunoprecipitation (IP) and Western Blot (WB). | | | | |
| --- | --- | --- | --- | --- |
| **Name** | **Gene** | **Amplicon** | **Sequence (5' > 3')** | **Applications** |
| *5-P21* | *P21/Cip1/Waf1* | 159 bp | CGGTGGAACTTTGACTTCGT | PCR |
| *3-P21* |  |  | CAGGGCAGAGGAAGTACTGG |  |
| *5-Cd36* | *Cd36* | 190 bp | tgctggagctgttattggtg | PCR |
| *3-Cd36* |  |  | tgggttttgcacatcaaaga |  |
| *5-B2m* | *B2m* | 177 bp | atgggaagccgaacatactg | PCR |
| *3-B2m* |  |  | cagtctcagtgggggtgaat |  |
| **Name** | **Company** | **Cat. No.** | **Epitope** | **Applications** |
| GFP | Roche | #1181446 | Tagged fusion | IP/WB/IF |
| P-ERK1/2 | Cell Signaling | #4370 | Phosphorylation (Y202/204) | WB |
| P-PPARγ | Abcam | #60953 | Phosphorylation (S82/112) | WB |
| PPARγ | Cell Signaling | #2435 | N-terminal | IF/IHC |
| PPARγ | Santa Cruz | #7196 | N-terminal | IP |
| MTMR7 | Santa Cruz | #51145 | Internal | IP |
| MTMR7 | Abcam | #121222 | Internal | IP/WB/IF |
| MTMR7 | Abcam | #150458 | C-terminal | IHC/WB |
| MTMR7 | MyBioSource | #9406043 | C-terminal | IHC |
| HSP90 | Santa Cruz | #7947 | Input/loading control | WB |
| lamin A/C | Santa Cruz | #20681 | Input/loading control | WB |
| β-tubulin | Abcam | #15568 | Input/loading control | WB |

| **Table S3 MM/GBSA Binding Free Energies ***  * Binding free energies (ΔG) and standard deviations calculated for PPARγ in complex with the peptide on the coactivator binding site or helix 11. For the calculation, the trajectories from the MD simulation were used, excluding the equilibration steps. As a reference, the binding free energy of RXRα to PPARγ was calculated. Legend: MM/GBSA = molecular mechanics/generalized Born and surface area continuum solvation. | | |
| --- | --- | --- |
| **Complex** | **PPARγ interaction site** | **ΔG [kcal/mol]** |
| PPARγ/RXRα | Helix 10 | *-46,35 ± 6,96* |
| PPARγ/PEP | Coactivator binding site | *-69,04 ± 7,04* |
| PPARγ/MP | Coactivator binding site | *-79,73 ± 8,24* |
| PPARγ/PEP | Helix 11 | *-39,85 ± 8,21* |

| **Table S4** **Listings of the pairs of interacting residues in the peptide/PPARγ complexes**.  Interactions calculated with AMBER18 for the trajectory of a 120 ns MD simulation with maximal atom distance set to 5 Å, excluding hydrogens. Interactions had to be present in more than 80 % of the 50.000 frames to be included. **A:** Interacting residue pairs with PEP bound to the coactivator interaction site of PPARγ. **B:** Interacting residue pairs with MP bound to the co-activator interaction site of PPARγ. **C:** Interacting residues between the Leucines of the LXXLL motif of SRC1 with the co-activator interaction site on PPARγ in *green*. Interactions calculated with AMBER18 for the crystal structure from 1fm9 (chain D and E) with maximal atom distance set to 5 Å, excluding hydrogens. Residues that were also predicted to be part of the interactions with PEP or MP are marked in *green* in A and B. **D:** Residues determined by Nolte et al. (Nature 1998; 395:137-143) to be part of the co-activator interaction site. Residues that were also predicted to be part of the interactions with PEP or MP are marked with a star (*) in A and B. |
| --- |
|  |
